# Comparative effectiveness of abatacept, apremilast, secukinumab and ustekinumab treatment of psoriatic arthritis: a systematic review and network meta-analysis

**DOI:** 10.1007/s00296-017-3919-7

**Published:** 2017-12-28

**Authors:** P. Kawalec, P. Holko, P. Moćko, A. Pilc

**Affiliations:** 1 Drug Management Department, Institute of Public Health, Faculty of Health Sciences, Jagiellonian University Collegium Medicum, Grzegorzecka 20, 31-531 Kraków, Poland; 2 Institute of Pharmacology Polish Academy of Sciences, Kraków, Poland

**Keywords:** Biologics, Biologic drugs, Pharmacotherapy, Network meta-analysis, Systematic review

## Abstract

To assess the comparative effectiveness and safety of novel biologic therapies in psoriatic arthritis (PsA) and to establish the position of the non-anti-tumor necrosis factor α (TNF-α) biologic drugs in the treatment regimen of the disease. A systematic review and network meta-analysis (NMA) was conducted according to the preferred reporting items for systematic reviews and meta-analyses (PRISMA) requirements. Two investigators identified the studies, abstracted data, and assessed the risk of bias independently. The NMA was conducted for efficacy [American College of Rheumatology (ACR) criteria, ACR20 and ACR50; psoriasis area and severity index (PASI), PASI75] and safety outcomes [any adverse events (AEs) and serious adverse events (SAEs)]; treatments were ranked using the *P* score for each outcome. The PROSPERO registration number was 42017072200. MEDLINE/PubMed, Embase, Cochrane Library, and ClinicalTrials.gov were searched from the inception of each database to July 10, 2017. Randomized controlled trials (RCTs) for abatacept, apremilast, secukinumab or ustekinumab in adults with moderate and severe PsA were included. The overall PsA population and anti-TNF-α-naive, anti-TNF-α-failure, or anti-TNF-α-experienced subpopulations were considered. We identified eight eligible RCTs and included them in the systematic review and NMA. Significant differences in ACR20 response rate were revealed between secukinumab 150 mg and apremilast 20 mg [relative risk; RR = 2.55 (CI—confidence interval; 1.24, 5.23)] and between secukinumab 300 mg and apremilast 20 or 30 mg [RR = 3.57 CI (1.48, 8.64) and RR = 2.84 CI (1.18, 6.86), respectively]. Any AEs occurred more often in apremilast 20 and 30 mg compared with placebo [RR = 0.58 CI (0.45, 0.74) and RR = 0.58 CI (0.45, 0.75), respectively] but also compared with secukinumab 150 mg [RR = 0.54 CI (0.35, 0.81) and RR = 0.45 CI (0.35, 0.82), respectively]. No significant differences were revealed for SAEs among biologics and between biologics and placebo. In the overall population, as well as in the anti-TNF-α-naive subpopulation, secukinumab at a dose of 300 and 150 mg was ranked the highest for the ACR20 endpoint, while in the anti-TNF-α-experienced subpopulation, secukinumab 300 mg and apremilast 30 mg revealed the highest rank. Secukinumab 75 mg was the safest drug in terms of any AEs, but for SEAs the safest was ustekinumab 90 mg. Our study revealed no significant differences among non-anti-TNF-α biologics in the treatment of PsA in the comparisons performed with regards to the highest efficacy and safety. Both in the overall population and in the analyzed subpopulations, secukinumab 300 mg was ranked the highest for the ACR20 response rate. Secukinumab 300 mg was the safest drug in terms of any AEs, and ustekinumab 90 mg presented the lowest overall risk of SAEs. Head-to-head trials and evaluation of comparative efficacy and safety between non-TNF-α biologics are warranted to inform clinical decision making with a relevant treatment paradigm.

**Electronic supplementary material:**

The online version of this article (10.1007/s00296-017-3919-7) contains supplementary material, which is available to authorized users.

## Introduction

Psoriatic arthritis (PsA) is a chronic autoimmune disease classified as a seronegative spondyloarthropathy; inflammatory process of the vertebral column, joints, fingers, and toes, as well as other arthropathies are the main symptoms of this condition. The disease has a significant impact on patients’ functioning and quality of life [1, 2]. PsA is associated with an increased morbidity and mortality, resulting in a reduced quality of life and productivity [3]. The prevalence of PsA is higher among patients with psoriasis, with skin lesions typically preceding joint inflammation by several years and a prevalence rate ranging from 7 to 26% [4, 5].

Conventional treatment for PsA usually begins from disease-modifying antirheumatic drugs (DMARDs) and nonsteroidal anti-inflammatory drugs (NSAIDs). If this treatment is not effective or patients are intolerant, other biologic therapies should be used. In the European Union, there are registered biologic drugs designed to block the activity of an inflammation cytokine, called tumor necrosis factor (anti-TNF)-α inhibitors (such as infliximab, adalimumab, golimumab, etanercept) [6]. Appropriate treatment is aimed at slowing down the disease progression and/or relieving the symptoms and achieving clinical remission [7]. In patients with intolerance or primary or secondary nonresponsiveness to anti-TNF-α, another class of biologic drugs should be administered. Several new biologic agents have emerged in recent years along with improvements in the understanding of the pathogenesis of PsA and have been claimed to be effective in randomized controlled trials (RCTs): abatacept, apremilast, secukinumab and ustekinumab [6].

Abatacept is a soluble, fully human fusion protein consisting of the extracellular domain of cytotoxic T-lymphocyte antigen 4 (CTLA-4) linked to a modified Fc portion of human IgG1. It selectively inhibits T-cell activation via competitive binding to CD80 or CD86 and decreases the serum levels of cytokines and inflammatory proteins implicated in the pathogenesis of PsA [8]. Apremilast is an oral phosphodiesterase-4 inhibitor that has been shown to regulate inflammatory mediator inhibiting the expression of inflammatory cytokines and increasing the expression of anti-inflammatory mediators such as interleukin (IL) 10 [9, 10]. Secukinumab is a human immunoglobulin-G1 monoclonal antibody that selectively binds to and neutralizes IL-17A. Increased levels of cells that produce IL-17A have been observed in the circulation, joints, and skin plaques of patients with PsA, and these levels have been shown to correlate with measures of disease activity [11–13]. Ustekinumab is a human immunoglobulin G1 that binds to the common p40 subunit shared by IL-12 and IL-23 involved in the pathogenesis of PsA [14].

The drugs were registered for therapy of PsA by the US Federal Drug and Food Administration (FDA) and the European Medicines Agency (EMA) [6]; ustekinumab was registered in 2013 by the FDA and in 2009 by the EMA, apremilast in 2014 by the FDA and in 2015 by the EMA, secukinumab in 2016 by the FDA and in 2015 by the EMA, and abatacept was authorized for the condition in July 2017 in the USA and in August 2017 by the EMA [6, 15, 16].

According to the summary of product characteristics, ustekinumab should be used for a period of 28 weeks; therefore, the revealed data for 24 weeks of follow-up were considered appropriate for the assessment of this drug [14]. Apremilast should be administered for a period of 24 weeks, thus the reference studies which provided results for this follow-up period were deemed adequate for evaluation [10]. According to the marketing authorization, secukinumab should be administered for 16 weeks; however, in some patients, it could be administered for a longer period. Therefore, the inclusion of data for 16–24 weeks was also considered adequate [13]. For abatacept, a period of up to 6 months (24–26 weeks) is required, thus the reference clinical trials proved relevant for a credible efficacy assessment [16].

Activity of the disease is usually presented with a number of parameters included in the American College of Rheumatology 20% improvement criteria (ACR20). ACR20 is defined by the following three conditions: at least 20% improvement in the number of tender joints (based on 68 joints), at least 20% improvement in the number of swollen joints (based on 66 joints), and at least 20% improvement in three of five additional domains (patient’s global assessment, physician’s global assessment, pain, disability measured by the health assessment questionnaire, and acute-phase reactant measured by C-reactive protein levels) [17].

Skin involvement was assessed using the psoriasis area and severity index (PASI). The PASI was used to assess the response of psoriasis, rated as 50% improvement (PASI50), 75% improvement (PASI75), and 95% improvement (PASI90). The PASI is defined as the degree of erythema, induration, and desquamation of skin lesions and the area affected with psoriasis [17].

A group of patients with PsA do not tolerate or do not have a satisfactory response to either NSAIDs, nor non-biologic DMARDs; about 25–50% of patients are non-responsive to anti-TNF-α drugs [18, 19]. Therefore, non-anti-TNF-α biologic agents have emerged as second-line therapy in such situations. However, the comparative efficacy of these agents remains unknown as head-to-head RCTs are not available.

No direct studies on relative effectiveness of any drugs in the therapy of patients with PsA have been published so far, so data on the relative efficacy of the novel agents (abatacept, apremilast, secukinumab, or ustekinumab) are still unavailable. Conventional meta-analysis is unable to resolve this issue owing to its incapability of comparing three or more treatments; therefore, it is difficult to integrate information on the relative efficacy of all tested regimens. On the other hand, a network meta-analysis (NMA) can provide a comprehensive and coherent set of comparisons based on available evidence.

In a recent NMA, Song and Lee [18] compared apremilast, secukinumab, and ustekinumab for induction of ACR20 response and safety profile (severe adverse events or SAEs rate) in the overall population included in clinical trials. They found no significant differences between treatments; however, the studies included were heterogeneous in terms of design and patients’ characteristics. For example, they included phase two studies with a shorter follow-up (12–14 weeks) in the NMA for a follow-up of 12–24 weeks, and reclassified some study arms (apremilast 40 mg was analyzed as 30 mg; ustekinumab 63 mg was analyzed as 90 mg). Moreover, they did not assess the treatments in subgroups differentiated by previous exposure to anti-TNFs and did not include abatacept. The present study was performed to fill this information gap.

The aim of our study was to assess the comparative efficacy and safety profile of considered biologic therapy regimens in adults with moderate to severe active PsA and provide meaningful information on the optimal treatment regimen in this condition. The efficacy of non-anti-TNF-α agents in patients with PsA not treated with anti-TNF-α (naive), patients previously treated with anti-TNF-α agents (experienced), and in subgroups with inadequate response or intolerance to anti-TNF-α (failure) was assessed.

## Materials and methods

### Data sources and searches

A systematic review was conducted according to the methods and recommendations from the PRISMA Extension Statement for Reporting of Systematic Reviews incorporating NMA [20] recommendations for conducting and interpreting the NMA (developed by the International Society for Pharmacoeconomics and Outcomes Research [ISPOR] Task Force [21] and Cipriani et al. [22]). The systematic review was registered in the PROSPERO database (registration number: 42017072200).

**Fig. 1:**
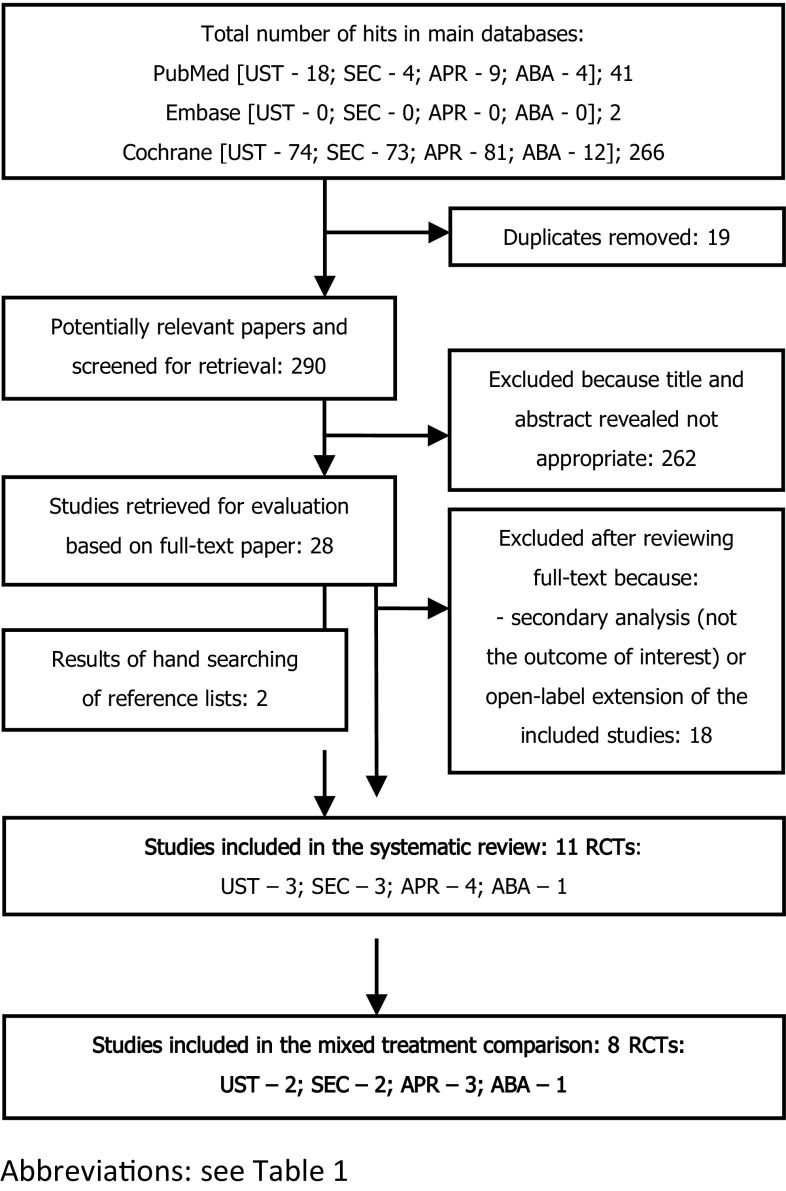
Search flow diagram

We identified eligible studies by searching MEDLINE via PubMed, Embase, and Cochrane Library. The search was performed from the inception of each database until July 10, 2017; studies published in English were considered. The search strategy was based on the medical subject heading (MeSH) terms or Emtree terms combined with Boolean logical operators (Table 1). The search strategy was presented with the quality of reporting of meta-analyses (QUOROM) diagram (Fig. 1). Ethical approval and informed consent were not required because our study did not collect information on patients or influence patient care. There were no restrictions with regard to endpoints, which allows to search for all possible adverse events, including severe, rare, and those which cannot be predicted prior to analysis. Similarly, refinements to the efficacy and effectiveness of the practice can be retrieved.

**Table 1:** Keywords used in the searches of medical databases to identify relevant studies for abatacept, apremilast, secukinumab, and ustekinumab for the treatment of patients with psoriatic arthritis

Area of meaning	Keywords
Health problem (population)	Psoriatic arthritis OR psoriasis arthritis OR psoriasis arthritic OR psoriatic arthropathy OR psoriatic arthropathies OR psoriatic polyarthritis OR Alibert Bazin disease
Intervention I (ABA)	Abatacept OR belatacept OR BMS224818 OR BMS-224818 OR BMS 224818 OR LEA29Y OR nulojix OR orencia OR BMS 188667 OR BMS-188667 OR BMS188667 OR CTLA-4-Ig OR CTLA4-Ig OR CTLA4-Fc
Intervention II (APR)	Apremilast OR otezla OR CC 10004 OR CC10004 OR CC-10004 OR small molecule
Intervention III (SEC)	Secukinumab OR cosentyx OR AIN 457 OR AIN457 OR AIN-457
Intervention IV (UST)	Ustekinumab OR stelara OR CNTO 1275 OR CNTO-1275 OR CNTO1275
Type of research	PubMed: humans, clinical trial, randomized controlled trial, comparative study, controlled clinical trial; embase: humans, only embase, controlled clinical trials, randomized controlled trial; cochrane: cochrane central register of controlled trials

*ABA* abatacept, *APR* apremilast, *CTLA-4* cytotoxic T-lymphocyte antigen 4, *UST* ustekinumab, *SEC* secukinumab

### Selection criteria

Studies were identified using the search strategy by two reviewers. Study selection was based on the title and abstract and, if necessary, full-text articles. Studies were selected for inclusion in this analysis based on the following criteria: (1) participants were defined as adults (18 years or older) with a clinical diagnosis of moderate to severe PsA; (2) intervention assessed was: abatacept, apremilast, secukinumab, and ustekinumab, and at least one study arm included a licensed dosage of those drug (EMA: [10, 13, 14, 16]); (3) comparator: another biologic agent or placebo; (4) outcomes: ACR20, ACR50, PASI75 (efficacy outcomes) and any AEs, SAEs, and withdrawals due to AEs (safety outcomes); (5) study: prospective, randomized trials with a follow-up of minimum 16 weeks but no longer than 28 weeks [maximum period for the assessment of response induction (EMA: [10, 13, 14, 16])]. We selected only those novel biologics which have been registered by the EMA in the European Union or by the FDA in the United States for the treatment of PsA and which are not included in the anti-TNF-α group. The efficacy (ACR20, ACR50, PASI75) and safety (any AEs, SAEs, withdrawal due to AEs) endpoints for the overall study population and each subpopulation (anti-TNF-α-naive, anti-TNF-α-failure, and anti-TNF-α-experienced) were included. The results among anti-TNF-α-experienced patients were used whenever information on anti-TNF-α-failure patients was not reported.

Full-text articles were included if they contained the required information about the study population, treatment regimen, and necessary data.

We excluded the following studies: (a) trials of novel (not registered in the European Union or USA) agents; (b) nonrandomized or uncontrolled studies; and (c) non-English publications. Unpublished studies, poster presentations, and conference abstracts were excluded if they did not provide information about the study design and results. Two study investigators independently reviewed the title and abstract of the studies identified in the search to exclude those that did not address the research question of interest based on prespecified inclusion and exclusion criteria.

The full text of the remaining articles was examined to determine whether it contained relevant information. Conflicts in study selection at this stage were resolved by consensus, referring to the original article. However, there was a high degree of compatibility between the reviewers (95%). We searched the bibliographies of these selected articles, systematic reviews, and clinical trial registries (http://www.clinicaltrials.gov) to identify any additional studies (hand searching).

### Data extraction and quality assessment

Data were also extracted independently by two reviewers using predefined data extraction forms. Studies that met the inclusion criteria were independently reviewed by two reviewers who extracted the following information: study design (methodology), participants’ characteristics, inclusion criteria, treatment regimen and concomitant therapy, duration of treatment, follow-up, sample size, arms size, and number of participants achieving predefined outcomes. The extracted data were then inspected by each author independently.

The intention-to-treat principle (ie, all patients lost to follow-up were considered treatment failures) was applied in the case of missing outcome data.

The methodological quality of eligible RCTs and the risk of bias within individual studies were assessed using the tool recommended by the Cochrane Collaboration, namely, domain-based evaluation. This tool allows to evaluate specific domains: sequence generation, allocation concealment, blinding of participants, blinding of outcome assessment, incomplete outcome data, selective outcome reporting, and “other issues”. We used the domain-based evaluation (“+”, low risk of bias; “−”, high risk of bias; “?”, unclear risk of bias) [23].

### Data analysis and synthesis

The NMA was conducted with R software netmeta package [24]. The package incorporates the graph-theoretical method of NMA (vertices—treatments, edges—randomized comparisons) and provides a point estimate from the network along with 95% confidence intervals (CIs). This frequentist method is an alternative to standard NMA conducted within the Bayesian framework [25].

In the NMA, we used consistency and random effects models with adjustment for multi-arm studies. All eligible treatment regimens with different doses and dosing intervals from the identified studies were included in the network and each treatment constituted one node (vertex in a graph). However, in the manuscript, only licensed dosage regimens were presented, that is, apremilast 30 mg, abatacept 10 mg, ustekinumab 45 mg, and secukinumab 150 mg (anti-TNF-α naive) or secukinumab 300 mg (anti-TNF-α failure, with plaque psoriasis). All comparisons, including suboptimal and experimental dosage regimens, were presented in supplementary file.

The heterogeneity of evidence was assessed using the *Q* test, *I* ^2^ statistic, and tau values, and consistency was assessed using the splitting approach and comparison with direct evidences. Publication bias was assessed by examining the funnel plot for “small-study effects”.

The ranking of the treatment was conducted using the *P* score, a frequentist equivalent of surface under the cumulative ranking or SUCRA. A higher *P* score corresponds to higher ranking for efficacy outcomes (ie, higher probability of ACR20, ACR50, etc) and higher ranking for safety [i.e., lower risk of adverse events (AEs)] [26]. The rankings for ACR20 and SAEs were combined in a Hasse diagram. Treatments in the upper section of the diagram are more efficacious (ACR20) and safer (SAEs) than the other treatments connected to them by arrows. Treatments not connected by arrows represent “mutually incomparable” ones, that is, treatments whose comparative order was different for SAEs and ACR20 (e.g., treatment A ranked higher for ACR20 but ranked lower for SAEs than treatment B) [27].

Caution should be noted when interpreting the treatment ranking alone since it informs only about probability of a treatment to be the best while not incorporating the effect size of the difference between treatments directly. The average probability of an event (or sample sizes of hypothetical studies) along with relative measures from NMA should be considered with the treatment rankings [20, 28, 29].

The NMA was conducted for odds ratio (OR). The OR was used to calculate the average probability of an event for each treatment, using the assumed probability in the control arm. The latter was obtained from the meta-analysis of placebo arms from all studies included in the NMA, using the random effects model based on the Freeman-Tukey (double arcsine) transformed proportion. The average probability of ACR20 response to each treatment was used to calculate sample sizes for hypothetical between-treatment comparative studies, using the likelihood-ratio test assuming 80% power and a two-sided alpha of 0.05.

A *P* value of less than 0.05 was considered statistically significant.

## Results

### Systematic review

The results of the systematic review are summarized in Fig. 1. In the systematic review, we revealed 11 RCTs presented in 11 publications [17, 30–39]. After careful consideration, we have chosen eight placebo-controlled studies referring to abatacept [30], apremilast, [31–33], secukinumab, [34, 35] and ustekinumab, [36, 37] homogeneous enough to be used in the NMA as well as providing results for the desired study endpoints in a 16- or 24-week follow-up period. Selected trials were excluded if they had an inadequate follow-up period limited to 12 weeks and/or a small study population [17, 38, 39].

The baseline characteristics of the studies as well as homogeneity assessment were summarized in Table S1. The baseline characteristics were generally similar between the studies, with a few exceptions. Some more heterogeneity was observed with regards to concomitant therapy, with methotrexate being allowed in most reference clinical trials. The majority of the included patients were treated with NSAIDs or DMARDs.

**Fig. 2:**
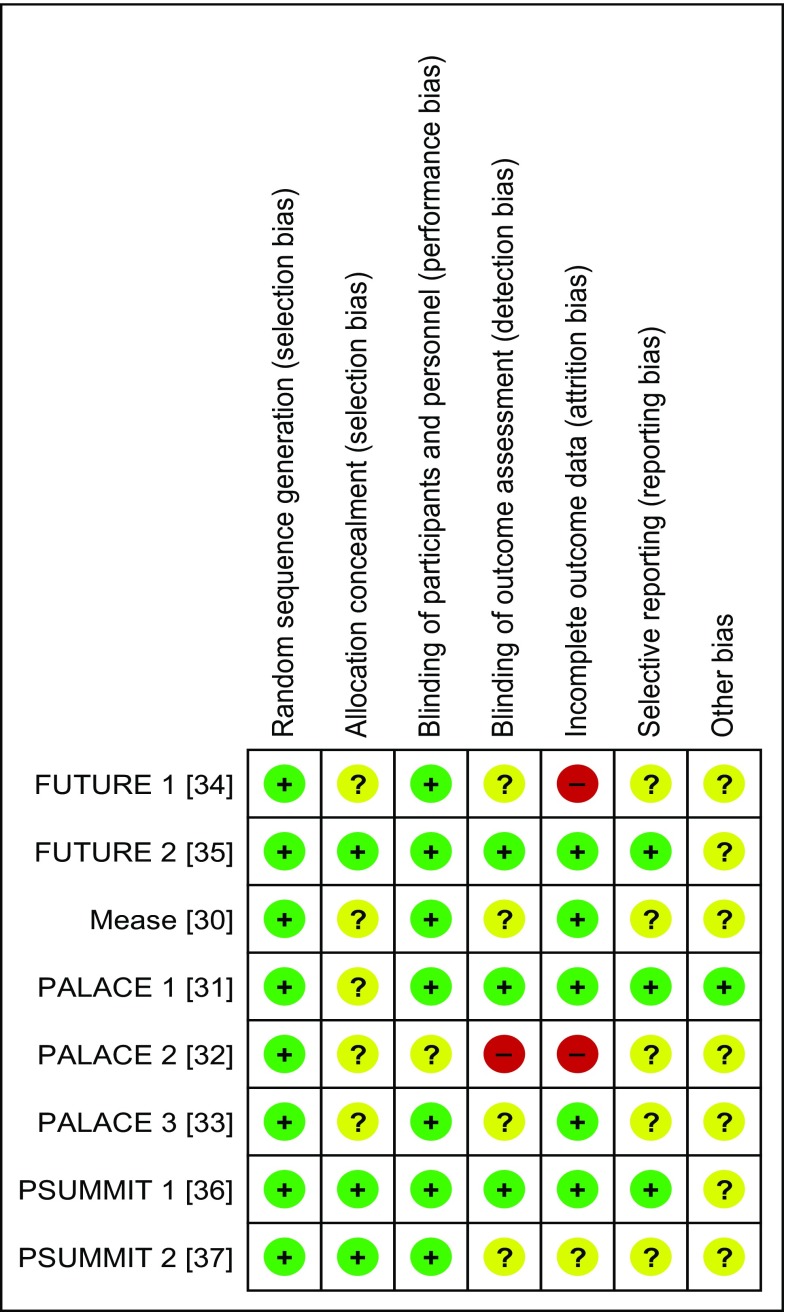
Risk of bias summary

The methodological quality of RCTs in this review was categorized as high, and the risk of bias was assessed as low. The probability of occurrence of bias in most studies and domains was considered low (Fig. 2). All the included studies were randomized and almost all were double-blind [30, 31, 33–37]. Three of the studies specified the method of randomization [35–37] and the other three included a description of the blinding method [31, 35, 36].

The analysis of the homogeneity of the included trials showed that the efficacy analysis, measured by ACR response, could be performed for ACR20 for three trials [31–33]. The analysis could also be performed for ACR50 for two trials [32, 33] at week 16 and for five trials [30, 31, 34, 35, 37] at week 24. The efficacy of psoriasis treatment based on PASI75 was assessed in two trials at week 16 [32, 33] and four trials [31, 34, 35, 37] at week 24.

The safety analysis was performed for any AEs, SAEs, and withdrawals due to AEs in six trials [30–33, 36, 37] for a follow-up period of 24 weeks, and in two trials [34, 35] for a follow-up period of 16 weeks.

In all eight reference studies, biologic drugs proved significantly more effective compared with placebo in terms of the ACR20 and ACR50 response rate [30–37].

### NMA results

Eight trials were homogeneous enough to perform an NMA for the overall population as well as for the anti-TNF-α-naive subpopulation [30–37]. Five studies were appropriate to perform an NMA for the anti-TNF-α-experienced subpopulation, [30–33, 37] and four were appropriate for inadequate response to anti-TNF therapy and/or discontinued treatment due to safety or tolerability issues (anti-TNF-α failure) [31, 33–35]. The results of abatacept treatment and ustekinumab treatment among anti-TNF-α-failure patients were not reported [30, 37]. Hence, data regarding anti-TNF-α-experienced patients were used in the base-case analyses for those drugs. Similarly, the results of secukinumab treatment among anti-TNF-α-experienced patients were not reported and the results among anti-TNF-α-failure patients were used [34, 35].

The follow-up period of the reference studies varied from 12 to 54 weeks, but the period of therapy administration incorporated into the dataset was a minimum of 16 weeks and did not exceed 24 weeks, as placebo control groups were available for this follow-up period. After 24 weeks of therapy, patients in the placebo groups were re-randomized and active therapy study without placebo control was continued/conducted.

#### Ranking of the treatments

The *P* score-based ranking of the treatments was presented in Table 2 (licensed dosages only) and in Table S2 in supplementary file (all dosage regimens from clinical trials).

**Table 2:** *P* score (overall rank based on *P* score among presented treatments)

	Abatacept	Apremilast	Secukinumab 150	Secukinumab 300	Ustekinumab
ACR20, overall population	0.639 (3)	0.407 (5)	0.815 (2)	0.932 (1)	0.510 (4)
ACR50, overall population	0.829 (1)	0.291 (5)	0.706 (3)	0.759 (2)	0.324 (4)
PASI75, overall population affected by psoriasis	0.323 (5)	0.386 (4)	0.604 (3)	0.794 (1)	0.789 (2)
Any AE, overall population	0.370 (4)	0.123 (5)	0.473 (2)	0.567 (1)	0.410 (3)
SAEs, overall population	0.320 (4)	0.489 (3)	0.561 (2)	0.273 (5)	0.594 (1)
Withdrawal due to AEs, overall population	0.462 (3)	0.076 (5)	0.629 (2)	0.420 (4)	0.794 (1)
ACR20, anti-TNF-α-naive patient population	0.712 (3)	0.354 (5)	0.867 (2)	0.899 (1)	0.380 (4)
ACR20, anti-TNF-α-failure population^a^	0.405 (5)	0.608 (2)	0.508 (4)	0.848 (1)	0.583 (3)
ACR20, anti-TNF-α-experienced population^b^	0.421 (5)	0.630 (2)	0.536 (4)	0.888 (1)	0.616 (3)

Only the dosages recommended by the EMA were presented

*ACR* American College of Rheumatology, *AE* adverse event, *PASI* Psoriasis Area and Severity Index, *SAE* severe adverse event

^a^ With supplementation of results from anti-TNF-α-experienced population for abatacept and ustekinumab

^b^With supplementation of results from anti–TNF-α-failure population for secukinumab

Considering ACR endpoints in the overall population and in the anti-TNF-α-naive subpopulation, secukinumab and abatacept were ranked higher than the other treatments, while among anti–TNF-α-failure and anti-TNF-α-experienced subpopulations, apremilast and secukinumab were the best options followed by ustekinumab. Ustekinumab and secukinumab 150 mg were assessed as the safest according to the *P* score for SAEs and withdrawals due to AEs.

**Fig. 3:**
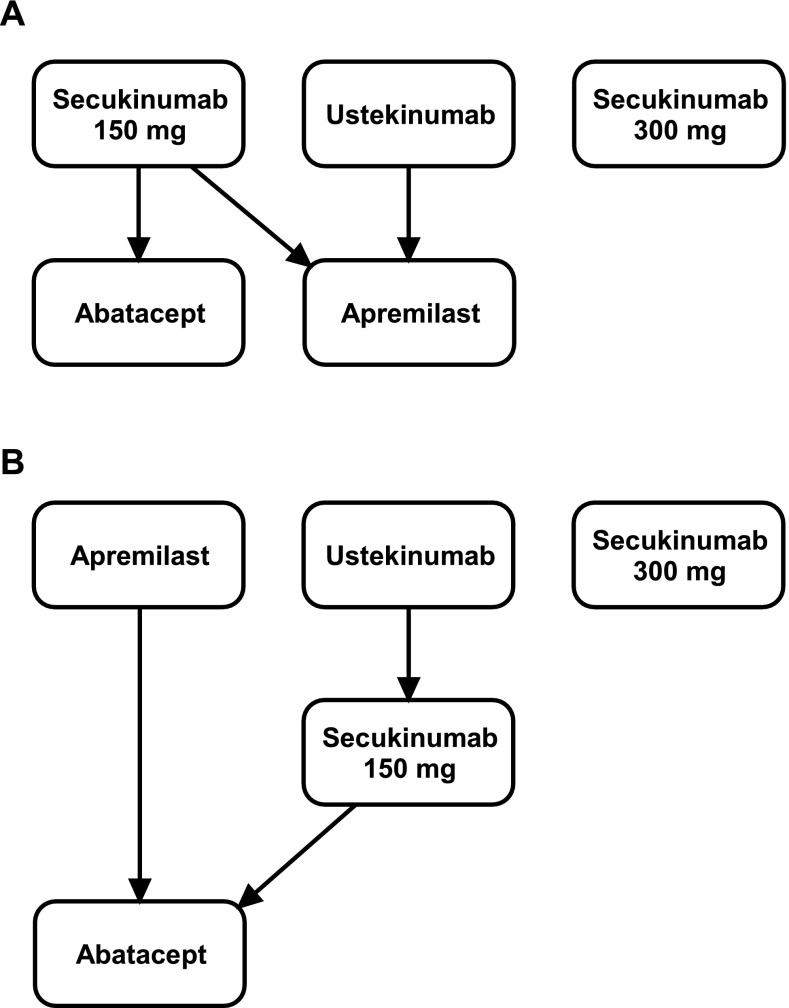
Hasse diagram combining *P* scores for ACR20 and SAEs among patients from the overall study population and anti-TNF-α-naive subpopulation (**a**), and anti-TNF-α-failure and anti-TNF-α-experienced subpopulation (**b**)

The combined ranking of ACR20 and SAEs among patients from the overall population and anti-TNF-α-naive subpopulation revealed that secukinumab and ustekinumab were the best treatments. Ustekinumab was not as efficacious as secukinumab, but was a safer option. Among anti-TNF-α-failure and anti-TNF-α-experienced subpopulations, apremilast and ustekinumab were the best treatment options. The use of secukinumab 300 mg was warranted within all populations along with the above options, but its low rank for SAEs did not allow a direct comparison with other treatments (Fig. 3).

#### Relative treatment effects

No significant differences between treatments were revealed with the exception of the following: (1) secukinumab 300 mg increased the ACR20 response rate in the overall population in comparison with apremilast (*P* = 0.020); (2) apremilast reduced the rate of withdrawal due to AEs in comparison with ustekinumab (*P* = 0.002); (3) secukinumab 150 and 300 mg increased the ACR20 response rate in the anti-TNF-α-naive subpopulation in comparison with apremilast and ustekinumab (*P* ranging from 0.004 to 0.024). There was no evidence for the higher efficacy of secukinumab over apremilast and/or ustekinumab in the anti-TNF-α-failure and anti-TNF-α-failure subpopulations (Table 3).

**Table 3:** Results of network meta-analyses: odds ratio with 95% confidence intervals in the brackets; in case of confidence intervals including 1 the difference is statistically non-significant

A	ABA_10	1.49 (0.44, 5.09)	**3.85 (1.22, 12.08)**	0.74 (0.21, 2.64)	0.53 (0.13, 2.08)	1.25 (0.30, 5.20)
	0.20 (0.02, 1.90)	APR_30	**2.58 (1.66, 4.01)**	0.49 (0.24, 1.01)	*0.35 (0.15, 0.85)*	0.84 (0.32, 2.18)
	**0.07 (0.01, 0.65)**	**0.36 (0.21, 0.63)**	PLC	**0.19 (0.11, 0.34)**	**0.14 (0.06, 0.29)**	**0.33 (0.14, 0.76)**
	0.50 (0.05, 4.84)	2.49 (1.07, 5.78)	**6.86 (3.64, 12.93)**	SEC_150	0.71 (0.35, 1.47)	1.70 (0.62, 4.70)
	0.59 (0.06, 6.01)	2.91 (1.08, 7.85)	**8.01 (3.52, 18.25)**	1.17 (0.57, 2.41)	SEC_300	2.39 (0.77, 7.44)
	0.21 (0.02, 2.43)	1.07 (0.32, 3.55)	**2.93 (1.01, 8.53)**	0.43 (0.12, 1.48)	0.37 (0.10, 1.41)	UST_45
B	ABA_10	0.67 (0.04, 10.39)	3.33 (0.25, 44.04)	0.34 (0.02, 5.44)	0.18 (0.01, 3.30)	0.17 (0.01, 3.35)
	1.24 (0.44, 3.49)	APR_30	**4.98 (1.97, 12.55)**	0.51 (0.13, 1.97)	0.27 (0.06, 1.35)	0.25 (0.04, 1.49)
	0.73 (0.27, 1.97)	**0.58 (0.45, 0.75)**	PLC	**0.10 (0.04, 0.27)**	**0.05 (0.01, 0.20)**	**0.05 (0.01, 0.23)**
	0.86 (0.30, 2.45)	0.69 (0.46, 1.04)	1.18 (0.85, 1.64)	SEC_150	0.53 (0.15, 1.83)	0.48 (0.08, 2.96)
	0.79 (0.26, 2.42)	0.64 (0.37, 1.11)	1.09 (0.67, 1.79)	0.93 (0.57, 1.52)	SEC_300	0.91 (0.12, 6.81)
	0.91 (0.32, 2.60)	0.73 (0.49, 1.10)	1.25 (0.91, 1.73)	1.06 (0.67, 1.68)	1.15 (0.63, 2.07)	UST_45
C	ABA_10	1.84 (0.18, 18.90)	1.80 (0.20, 16.09)	2.10 (0.18, 23.99)	1.04 (0.07, 14.65)	2.33 (0.19, 28.77)
	2.56 (0.37, 17.60)	APR_30	0.98 (0.45, 2.14)	1.14 (0.31, 4.26)	0.57 (0.11, 3.01)	1.26 (0.29, 5.44)
	1.46 (0.23, 9.24)	**0.57 (0.33, 1.00)**	PLC	1.17 (0.41, 3.37)	0.58 (0.13, 2.54)	1.30 (0.38, 4.44)
	0.66 (0.07, 6.28)	0.26 (0.06, 1.05)	0.45 (0.13, 1.64)	SEC_150	0.50 (0.11, 2.34)	1.11 (0.22, 5.61)
	1.15 (0.10, 12.69)	0.45 (0.09, 2.32)	0.78 (0.17, 3.67)	1.73 (0.27, 10.92)	SEC_300	2.23 (0.33, 15.26)
	0.40 (0.05, 3.31)	*0.16 (0.05, 0.50)*	**0.28 (0.10, 0.76)**	0.61 (0.12, 3.12)	0.35 (0.06, 2.23)	UST_45
D	ABA_10	2.07 (0.55, 7.76)	**5.00 (1.43, 17.53)**	0.74 (0.19, 2.92)	0.65 (0.15, 2.85)	1.93 (0.49, 7.51)
	1.76 (0.14, 22.89)	APR_30	**2.41 (1.60, 3.64)**	*0.36 (0.18, 0.72)*	*0.31 (0.13, 0.76)*	0.93 (0.48, 1.81)
	0.45 (0.07, 3.07)	0.26 (0.05, 1.40)	PLC	**0.15 (0.08, 0.26)**	**0.13 (0.06, 0.28)**	**0.39 (0.23, 0.65)**
	1.32 (0.17, 10.15)	0.75 (0.12, 4.68)	**2.92 (1.46, 5.85)**	SEC_150	0.88 (0.42, 1.85)	*2.61 (1.21, 5.65)*
	2.96 (0.34, 25.34)	1.68 (0.24, 11.83)	**6.57 (2.51, 17.19)**	2.25 (0.92, 5.46)	SEC_300	*2.97 (1.15, 7.62)*
	1.53 (0.19, 12.70)	0.87 (0.13, 5.91)	**3.41 (1.41, 8.22)**	1.17 (0.38, 3.58)	0.52 (0.14, 1.91)	UST_45
E	ABA_10	0.64 (0.08, 5.37)	2.22 (0.33, 15.18)	0.76 (0.10, 5.86)	0.34 (0.04, 2.90)	0.65 (0.08, 5.40)
		APR_30	3.47 (1.40, 8.59)	1.19 (0.38, 3.72)	0.53 (0.14, 1.98)	1.02 (0.29, 3.60)
			PLC	**0.34 (0.17, 0.68)**	**0.15 (0.06, 0.40)**	**0.29 (0.12, 0.71)**
				SEC_150	0.45 (0.18, 1.08)	0.86 (0.28, 2.63)
					SEC_300	1.93 (0.52, 7.10)
						UST_45

(A) ACR20, overall population (above treatments labels; *I* ^2^ = 56.7%) and ACR50, overall population (below labels; *I* ^2^ = 38.3%); (B) PASI75, overall population (above labels; *I* ^2^ = 59.4%) and any adverse events (below labels; *I* ^2^ = 0%); (C) severe adverse events (above labels; *I* ^2^ = 21.2%) and withdrawal due to adverse events (below labels; *I* ^2^ = 0%); (D) ACR20, anti-TNF-α-naive subpopulation (above labels; *I* ^2^ = 35.6%) and ACR20, anti-TNF-α-failure subpopulation (below labels; *I* ^2^ = 0%); (E) ACR20, anti-TNF-α-failure subpopulation (above labels; *I* ^2^ = 0%)

PLC, placebo; others, see Tables 1 and 2; OR > 1 below treatment labels means that the top treatment increases the odds for an outcome in comparison to the right one; OR > 1 above treatment labels means that the left treatment increases the odds for an outcome in comparison to the bottom one. The treatment is better when increases odds for efficacy outcomes or decreases the odds for safety outcomes in comparison to the other

Bolded values indicate a statistically significant Odds Ratios for comparisons with placebo

Italicized values indicate a statistically significant Odds Ratios in comparisons with active comparators (other considered biologic)

Compared with placebo, all treatments induced a higher rate of ACR20 and ACR50 responses in the overall population. All treatments except abatacept significantly increased the rate of PASI75 response compared with placebo. Only apremilast reduced the rate of any AEs and SAEs in comparison with placebo. Ustekinumab was the only treatment which significantly increased the rate of withdrawal due to AEs compared with control.

Abatacept and apremilast were no better than placebo in inducing ACR20 response among patients from the anti-TNF-α-failure and anti-TNF-α-experienced subpopulations.

#### Sample sizes of hypothetical studies

**Fig. 4:**
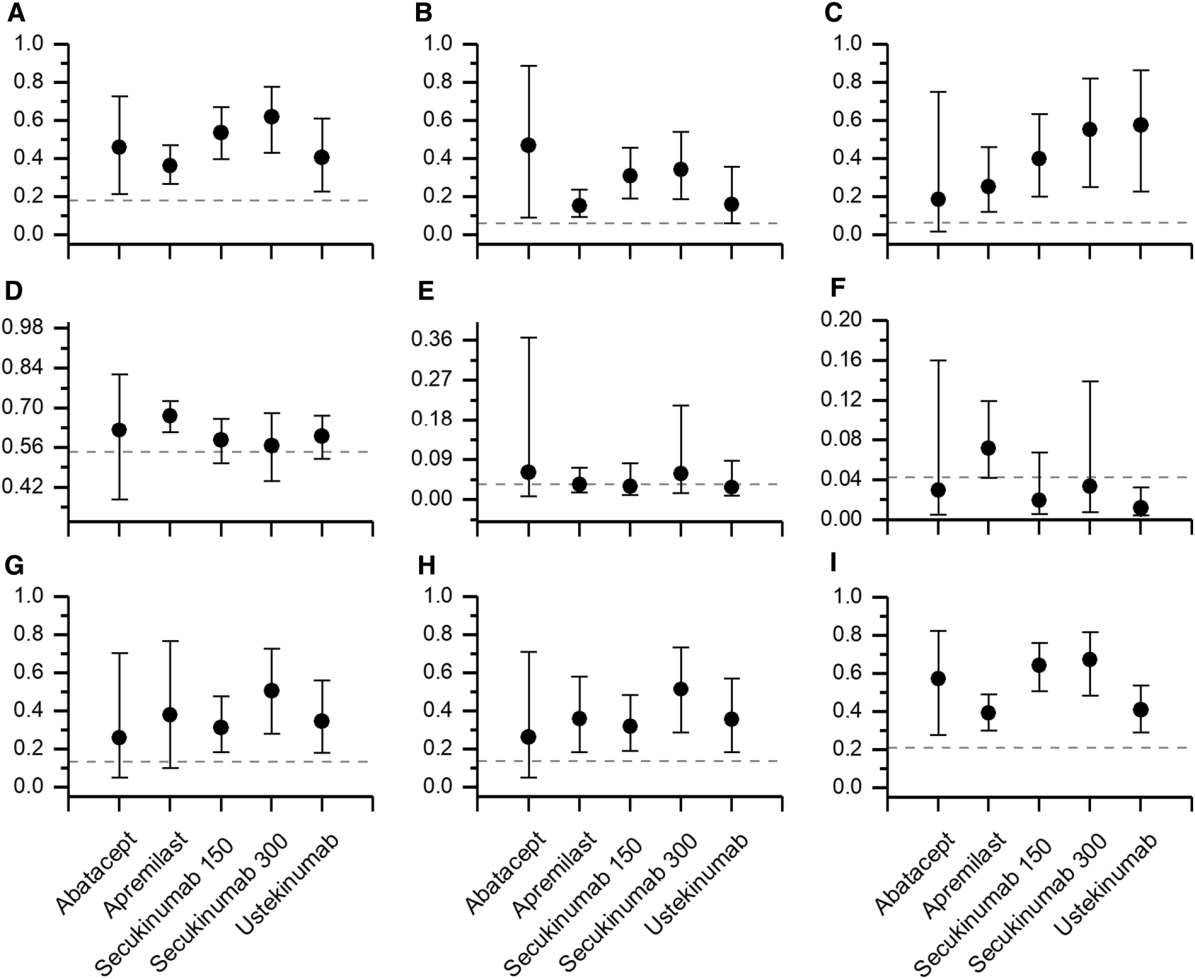
The average probability of **a** ACR20, overall population; **b** ACR50, overall population; **c** PASI75, overall population; **d** any adverse events; **e** severe adverse events; **f** withdrawals due to adverse events; **g** ACR20, anti-TNF-α-failure subpopulation; **h** ACR20, anti-TNF-α-experienced subpopulation; **i** ACR20, anti-TNF-α-naive subpopulation. Dashed line indicates assumed control rate (meta-analysis of placebo arms)

Apart from comparing secukinumab with apremilast and ustekinumab in the anti-TNF-α-naive subpopulation, the NMA models for ACR20 revealed no significant additional clinical benefit of any active treatment over the other. However, the magnitude of differences in point estimates of the average response rates (Fig. 4) may suggest clinical advantage of some treatments over the others, provided that the differences were not random or biased due to, for example, difference in trials’ design or participants’ characteristics.

The sample sizes of theoretical studies powered to prove the observed difference in ACR20 response between treatments are presented in Table S6 in supplementary file (all dosage regimens from clinical trials) and in Table 4 (licensed dosage regimens).

**Table 4:** Total sample sizes of comparative efficacy studies with participants from anti-TNF-α-naive subpopulation (above treatment labels) and anti-TNF-α-failure subpopulation (below treatment labels)

Abatacept	242	1432	724	296
460	Apremilast	122	98	24,938
NA	NA	Secukinumab 150	8564	142
120	480	NA	Secukinumab 300	112
822	7176	NA	304	Ustekinumab

The recommended dosage of the treatments is included

NA, not applicable (only secukinumab 300 mg is recommended in anti–TNF-α-failure population)

Only studies comparing secukinumab with apremilast or ustekinumab should include a relatively small number of participants from the anti-TNF-α-naive subpopulation (below 150 patients). Other comparisons would require much higher sample sizes from the anti-TNF-α-naive subpopulation, ranging from 242 to 25,000 patients, which makes them much less probable to conduct. Among anti-TNF-α-failure patients, the sample sizes for the comparison of secukinumab 300 mg with abatacept and ustekinumab were reasonable (120 and 304 patients, respectively).

#### Assessment of the networks

The level of heterogeneity of the effect sizes was high in the networks assessing response rates in the overall patient populations and anti-TNF-α-naive subpopulation (*I* ^2^ ranging from 35.6 to 59.4%), while in the other networks level of heterogeneity did not exceed 21.2%.

The evidence for secukinumab (mainly different response rates for secukinumab 75 mg) was a major contribution to observed heterogeneity of the networks. Because secukinumab studies differed in design (four-arm vs. three-arm studies), different efficacy resulted in significant between-design heterogeneity within the networks for ACR20 (overall population, *P* = 0.026; anti-TNF-α-naive population, *P* = 0.034) and PASI75 (*P* = 0.002).

The splitting approach revealed nonsignificant disagreement between direct and indirect evidences within all networks except for the comparison of PASI75 response between secukinumab 300 mg and placebo (*P* = 0.045) and between secukinumab 75 and 150 mg (*P* = 0.028). Overall, evidences for secukinumab were attributed with the greatest disagreement.

The results of all NMA models were convergent with ORs calculated for each study separately and the results of meta-analyses of direct evidences. There was no evidence of publication bias in any of the networks.

## Discussion

Although RCTs provide the best evidence for the relative efficacy of drugs, we did not identify such trials for non-anti-TNF-α agents in PsA therapy. We also revealed no prospective observational studies available, so our study provided the first insights on the comparative efficacy of those drugs in relation with previous exposure to anti-TNF-α agents. In the absence of head-to-head trials, the results may be useful to facilitate relevant decision making in terms of medical management and may inform a proper and effective selection of biologics in the management of active PsA in clinical guidelines and everyday practice.

Combining direct and indirect evidence from eight reference clinical trials, including anti-TNF-α-naive, anti-TNF-α-failure, or anti-TNF-α-experienced patients, allowed us to make several key observations.

Significant differences in the ACR20 response rate were revealed between secukinumab 150 mg and apremilast 20 mg and between secukinumab 300 mg and apremilast 20 or 30 mg. Any AEs occurred more often in apremilast 20 and 30 mg compared with placebo, but also compared with secukinumab 150 mg. No significant differences were revealed for SAEs among biologics and in the comparison of biologics with placebo.

In the overall population of PsA patients, it was confirmed that all approved agents, excluding abatacept 3 mg, are effective over placebo. Secukinumab 300 mg, secukinumab 150 mg, and abatacept were ranked the highest for the ACR20 response rate assessment in 16–24 weeks of follow-up. In the analyzed subpopulations, secukinumab 300 mg was ranked the highest for the ACR20 response rate, while in the anti-TNF-α-experienced subpopulation, secukinumab 300 mg and apremilast 30 mg had the highest rank.

The safest in terms of any AEs were secukinumab 75 mg and abatacept 30/10 mg. *P* score comparison for both ACR20 and SAEs revealed the highest rank for secukinumab 300 mg, ustekinumab, and secukinumab 150 mg. Apremilast was also ranked high for the anti-TNF-α-failure and anti-TNF-α-experienced subpopulations.

Our results would be especially valid in the case of patients who had persistently active disease despite traditional NSAID and/or DMARD therapy, or who do not tolerate NSAIDs and/or DMARDs, or those who were nonresponsive to anti-TNF-α therapy. Based on current data, the probability of failure to achieve ACR20 response in anti-TNF-α trials range from 25 to 50% [40–42]. In the case of such patients, the results of this innovative analysis are especially valid and clinically meaningful.

In a review of medical databases, we identified papers referring to the assessment of non-anti-TNF-α agents in PsA, but no study among PsA patients differentiated by previous anti-TNF-α treatment has been performed to date.

In a study by Ungprasert et al. [43], a total of 12 RCTs were identified and included in data analyses. Patients who received older anti-TNF-α (etanercept, infliximab, adalimumab, and golimumab) had a significantly higher chance of achieving ACR20 response compared with those receiving apremilast, ustekinumab, and certolizumab. The authors also revealed that the likelihood of achieving ACR20 response among secukinumab users (at a dose of 150 and 300 mg weekly) was significantly higher compared with apremilast or ustekinumab users, and the likelihood of achieving ACR20 response was higher among secukinumab users (at a dose of 150 and 300 mg weekly) compared with apremilast, ustekinumab, and certolizumab, even though the difference did not reach significance in a few comparisons (secukinumab 300 mg vs certolizumab, secukinumab 150 mg vs ustekinumab 90 mg, and secukinumab 300 mg vs ustekinumab 90 mg) [43]. The results of higher efficacy in terms of the ACR20 assessment were consistent with our findings suggesting the higher clinical effectiveness of secukinumab compared with other non-anti-TNF-α biologics; no safety profile comparisons between included drugs were performed.

In another study [44], a meta-analysis of abatacept, apremilast, secukinumab, and ustekinumab was performed. Two relevant studies were found to evaluate ustekinumab. The pooled results showed that the RRs for ACR20 versus placebo were 2.17 (95% CI 1.71–2.76) and 1.95 (95% CI 1.52–2.50) for ustekinumab 90 mg and ustekinumab 45 mg, respectively. A meta-analysis across the trials resulted in RRs for ACR20 versus placebo of 3.31 (2.04–5.36) for secukinumab 300 mg, 5.82 (1.56–21.71) for secukinumab 150 mg, and 4.47 (0.66–30.26) for secukinumab 75 mg. A meta-analysis resulted in RRs for apremilast 30 mg and apremilast 20 mg versus placebo of 1.98 (1.64–2.38) and 1.70 (1.40–2.06), respectively [44]. No relative assessments among the considered drugs were performed. Biologics have also demonstrated relatively good safety on a short as well as medium follow-up and no major safety issues were observed [44].

In another study [19], all four non-anti-TNF-α biologics were compared relatively among anti-TNF-α-experienced patients. There were no significantly different odds of achieving ACR20 response between those four agents in any studied dosages. These conclusions were generally in line with our findings.

The NMA models indicated a potential additional clinical benefit of secukinumab, abatacept, and apremilast dosages recommended by the EMA over the lower one used in the studies. Moreover, they indicated no or a small additional benefit of using ustekinumab 90 mg instead of ustekinumab 45 mg, which is also in line with the recommended dosage regimen.

It seems that the less strict approach to assess the homogeneity of studies included in the NMA used by Song and Lee [18] resulted in efficacy and dose–response relationship of apremilast, secukinumab, and ustekinumab. The conclusion for apremilast and secukinumab is in line with our findings. However, this study (Table S2 in supplementary file) as well as EMA recommendations did not indicate superior efficacy or safety profile of ustekinumab 90 mg over 45 mg. Additionally, they found no significant difference in the induction of ACR20 response between secukinumab 300 mg and apremilast 30 mg (OR 2.49; 95% Bayesian credible interval [CrI], 0.87, 6.64), but significant difference between secukinumab 300 mg and apremilast 20 mg (OR, 3.24; 95% CrI, 1.10, 8.37) was found. This study provided similar point estimates of the OR for the above comparisons (OR, 2.84; 95% CI, 1.18, 6.86 and OR, 3.57; 95% CI, 1.48, 8.64, respectively), but revealed significance for both. Song and Lee included a study by Schett et al. [39] in their models, and reclassified the apremilast arm of that study, assuming that a dose of 40 mg will have equal efficacy as that of 30 mg. The inclusion of that evidence despite lower credibility (phase II trial, no licensed dosage) resulted in an increase of the number of evidences in the network (precision of the estimates) and the average response rate for apremilast 30 mg. Hence, no significance of the results of the comparison with secukinumab was shown. The number of serious adverse events did not differ significantly among apremilast, secukinumab, ustekinumab and placebo; no significant differences were revealed in the safety between the interventions at different doses; conclusions occured the same as in our study. The ranking probability based on SUCRA indicated that ustekinumab 90 mg had the highest probability of being the most tolerable treatment followed by ustekinumab 45 mg [18].

Due to study limitations and differences between the compared studies, the interpretations should be considered with caution.

The limitations of the present study must be considered. First, the follow-up times ranged from 16 to 24 weeks, and were of medium duration. This period may be too short to evaluate the long-term effects. Thus, the results may not apply to long-term treatment but only to the response-induction phase of the treatment. The results may give meaningful information on the rate of patients with a clinical indication for long-term treatment.

Another issue is the comparability of the studies used for comparisons. The key issue for any indirect comparisons is the similarity of the patient populations. The studies included in the NMA were considered to be comparable in terms of the most important and essential parameters. The RCTs were homogenous in terms of the baseline characteristics of patients (similar female-to-male ratio, average age, and baseline disease activity as reflected by a similar patient’s global assessment of disease activity scores) and follow-up periods with outcomes reported. The definitions of active disease were consistent across the studies (ie, ≥ 3 swollen joints and ≥ 3 tender joints) and all studies allowed concomitant use of stable doses of DMARDs and steroids. On the other hand, the inevitable differences between the study characteristics could affect the results of indirect comparisons to some extent.

The available evidence for abatacept is limited. Only one phase II study with 40 participants per group met the inclusion criteria. Due to the small sample size and low number of evidence, the precision of results for abatacept treatment was relatively low and there was less credibility in comparison with other treatments.

Findings from the NMA need to be interpreted with caution since these trials did not always mirror clinical practice. Direct studies are needed to determine the relative efficacy and safety of the considered drugs.

The comprehensiveness of data comparisons is the major strength of this study, as this indirect comparison technique allows us to compare various pairs of biological agents for which direct comparison data are not available. We also exclusively included only RCTs that are considered as the most valid study design to prove the efficacy of an intervention. Thus, the primary data included in these analyses were of high quality. Another strength of this study was the methodological level with which it was conducted and the useful information it provided for clinicians and for healthcare decision makers.

In the protocol registered at PROSPERO also alefacept and brodalumab were presented but in the current stage of the project four biologics authorized both by FDA and EMA in PsA therapy were included. We have focused on ACR20 as primary and the most important outcome evaluated but results for other outcomes (ACR70 and ACR50) should also be presented on further steps of the project.

Our study revealed no significant differences among non–anti-TNF-α biologics in the treatment of PsA in the comparisons performed with regards to the highest efficacy and safety. Both in the overall population and in the analyzed subpopulations, secukinumab 300 mg was ranked the highest for the ACR20 response rate. Secukinumab 300 mg was the safest drug in terms of any AEs, and ustekinumab 90 mg presented the lowest overall risk of SAEs. Long-term studies based on a large number patient groups are needed to determine the relative efficacy and safety of abatacept, apremilast, secukinumab, and ustekinumab in PsA.

## Electronic supplementary material

Below is the link to the electronic supplementary material.

Supplementary material 1 (DOCX 81 KB)

Supplementary material 2 (DOCX 104 KB)

## References

[1] Day MS, Nam D, Goodman S (2012). Psoriatic arthritis. J Am Acad Orthop Surg.

[2] Rosen CF, Mussani F, Chandran V (2012). Patients with psoriatic arthritis have worse quality of life than those with psoriasis alone. Rheumatology.

[3] Walsh JA, McFadden ML, Morgan MD (2014). Work productivity loss and fatigue in psoriatic arthritis. J Rheumatol.

[4] Prey S, Paul C, Bronsard V (2010). Assessment of risk of psoriatic arthritis in patients with plaque psoriasis: a systematic review of the literature. J Eur Acad Dermatol Venereol.

[5] Radtke MA, Reich K, Blome C (2009). Prevalence and clinical features of psoriatic arthritis and joint complaints in 2009 patients with psoriasis: results of a German national survey. J Eur Acad Dermatol Venereol.

[6] European public assessment reports. http://www.ema.europa.eu/ema/index.jsp?curl=pages%2Fmedicines%2Flanding%2Fepar_search.jsp&mid=WC0b01ac058001d124&searchTab=&alreadyLoaded=true&isNewQuery=true&status=Authorised&status=Withdrawn&keyword=eksterminuja%CC%A8c&searchType=name&taxonomyPath=Diseases.Musculoskeletal+Diseases.Joint+Diseases.Arthritis&treeNumber=¤tCategory=Arthritis%2C+Psoriatic&searchGenericType=generics. Accessed 1 Sept 2017

[7] Gossec L, Smolen JS, Ramiro S (2015). European league against rheumatism (EULAR) recommendations for the management of psoriatic arthritis with pharmacological therapies: update. Ann Rheum Dis 2016.

[8] Weisman MH, Durez P, Hallegua D (2006). Reduction of inflammatory biomarker response by abatacept in treatment of rheumatoid arthritis. J Rheumatol.

[9] Schafer P (2012). Apremilast mechanism of action and application to psoriasis and psoriatic arthritis. Biochem Pharmacol.

[10] Otezla SmPC. https://ec.europa.eu/health/documents/community-register/2015/20150115130395/anx_130395_en.pdf. Accessed 1 Sept 2017

[11] Jandus C, Bioley G, Rivals JP (2008). Increased numbers of circulating polyfunctional Th17 memory cells in patients with seronegative spondyloarthritides. Arthritis Rheum.

[12] Noordenbos T, Yeremenko N, Gofita I (2012). Interleukin-17-positive mast cells contribute to synovial inflammation in spondylarthritis. Arthritis Rheum.

[13] Cosentyx SmPC. https://ec.europa.eu/health/documents/community-register/2015/20150115130444/anx_130444_en.pdf. Accessed 1 Sept 2017

[14] Stelara SmPC. http://www.ema.europa.eu/docs/en_GB/document_library/EPAR_-_Product_Information/human/000958/WC500058513.pdf. Accessed 1 Sept 2017

[15] Development History and FDA Approval Process for Orencia. https://www.drugs.com/history/orencia.html. Accessed 1 Sept 2017

[16] Orencia SmPC. http://www.ema.europa.eu/docs/en_GB/document_library/EPAR_-_Product_Information/human/000701/WC500048935.pdf. Accessed 1 Sept 2017

[17] McInnes IB, Sieper J, Braun J (2014). Efficacy and safety of secukinumab, a fully human anti-interleukin-17A monoclonal antibody, in patients with moderate-to-severe psoriatic arthritis: a 24-week, randomised, double-blind, placebo-controlled,phase II proof-of-concept trial. Ann Rheum Dis.

[18] Song GG, Lee YH (2017). Relative efficacy and safety of apremilast, secukinumab, and ustekinumab for the treatment of psoriatic arthritis. Z Rheumatol.

[19] Ungprasert P, Thongprayoon C, Davis JM (2016). Indirect comparisons of the efficacy of subsequent biological agents in patients with psoriatic arthritis with an inadequate response to tumor necrosis factor inhibitors: a meta-analysis. Clin Rheumatol.

[20] Hutton B, Salanti G, Caldwell DM (2015). The PRISMA extension statement for reporting of systematic reviews incorporating network meta-analyses of health care interventions: checklist and explanations. Ann Intern Med.

[21] Jansen JP, Trikalinos T, Cappelleri JC (2014). Indirect treatment comparison/network meta-analysis study questionnaire to assess relevance and credibility to inform health care decision making: an ISPOR-AMCP-NPC good practice task force report. Value Health.

[22] Cipriani A, Higgins JP, Geddes JR (2013). Conceptual and technical challenges in network meta-analysis. Ann Intern Med.

[23] Higgins JPT, Green S (2011). Cochrane handbook for systematic reviews of interventions version 5.1.0 [updated March 2011]. The Cochrane Collaboration. http://www.cochrane-handbook.org. Accessed 1 Sept 2017

[24] Rücker G (2012). Network meta-analysis, electrical networks and graph theory. Res Synth Methods.

[25] Neupane B, Richer D, Bonner AJ (2014). Network meta-analysis using R: a review of currently available automated packages. PLoS One.

[26] Rücker G, Schwarzer G (2015). Ranking treatments in frequentist network meta-analysis works without resampling methods. BMC Med Res Methodol.

[27] Carlsen L, Bruggemann R (2014). Partial order methodology: a valuable tool in chemometrics. J Chemom.

[28] Bhatnagar N, Lakshmi PV, Jeyashree K (2014). Multiple treatment and indirect treatment comparisons: an overview of network metaanalysis. Perspect Clin Res.

[29] Mavridis D, Giannatsi M, Cipriani A, Salanti G (2015). A primer on network meta-analysis with emphasis on mental health. Evid Based Ment Health.

[30] Mease P, Genovese MC, Gladstein G (2011). Abatacept in the treatment of patients with psoriatic arthritis: results of a six-month, multicenter, randomized, double-blind, placebo-controlled, phase II trial. Arthritis Rheum.

[31] Kavanaugh A, Mease PJ, Gomez-Reino JJ (2014). Treatment of psoriatic arthritis in a phase 3 randomised, placebo-controlled trial with apremilast, an oral phosphodiesterase 4 inhibitor. Ann Rheum Dis.

[32] Cutolo M, Myerson GE, Fleischmann RM (2016). A phase III, randomized, controlled trial of apremilast in patients with psoriatic arthritis: results of the PALACE 2 Trial. J Rheumatol.

[33] Blanco FB, Crowley J, Edwards ChJ (2016). Apremilast, an oral phosphodiesterase 4 inhibitor, in patients with psoriatic arthritis and current skin involvement: a phase III, randomised, controlled trial (PALACE 3). Ann Rheum Dis.

[34] Mease PJ, McInnes IB, Kirkham B (2015). Secukinumab Inhibition of Interleukin-17A in patients with psoriatic arthritis. N Engl J Med.

[35] McInnes IB, Mease PJ, Kirkham B (2015). Secukinumab, a human anti-interleukin-17A monoclonal antibody, in patients with psoriatic arthritis (FUTURE 2): a randomised, double-blind, placebo-controlled, phase 3 trial. Lancet.

[36] McInnes IB, Kavanaugh A, Gottlieb AB (2013). Efficacy and safety of ustekinumab in patients with active psoriatic arthritis: 1 year results of the phase 3, multicentre, double-blind, placebo-controlled PSUMMIT 1 trial. Lancet.

[37] Ritchlin C, Rahman P, Kavanaugh A (2014). Efficacy and safety of the anti-IL-12/23 p40 monoclonal antibody, ustekinumab, in patients with active psoriatic arthritis despite conventional non-biological and biological anti-tumour necrosis factor therapy: 6-month and 1-year results of the phase 3, multicentre, double-blind, placebocontrolled, randomised PSUMMIT 2 trial. Ann Rheum Dis.

[38] Gottlieb A, Menter A, Mendelsohn A (2009). Ustekinumab, a human interleukin 12/23 monoclonal antibody, for psoriatic arthritis: randomised, double-blind, placebo-controlled, crossover trial. Lancet.

[39] Schett G, Wollenhaupt J, Papp K (2012). Oral apremilast in the treatment of active psoriatic arthritis results of a multicenter, randomized, double-blind, placebo-controlled study. Arthritis Rheum.

[40] Antoni CE, Kavanaugh A, Kirkham B (2005). Sustained benefits of infliximab therapy for dermatologic and articular manifestations of psoriatic arthritis: results from the infliximab multinational PsA controlled trial (IMPACT). Arthritis Rheum.

[41] Genovese MC, Mease PJ, Thomson GT (2007). Safety and efficacy of adalimumab in treatment of patients with PsA who had failed disease modifying antirheumatic drug therapy. J Rheumatol.

[42] Mease PJ, Kivitz AJ, Burch FX (2004). Etanercept treatment of psoriatic arthritis: safety, efficacy, and effect on disease progression. Arthritis Rheum;.

[43] Ungprasert P, Thongprayoon C, Davis J (2016). Indirect comparisons of the efficacy of biological agents in patients with PsA with an inadequate response to traditional disease-modifying anti-rheumatic drugs or to non-steroidal anti-inflammatory drugs: a meta-analysis. Semin Arthritis Rheum.

[44] Ramiro S, Smolen JS, Landewé R (2016). Pharmacological treatment of psoriatic arthritis: a systematic literature review for the 2015 update of the EULAR recommendations for the management of psoriatic arthritis. Ann Rheum Dis.

