# Supplementary material for: Comparative effectiveness of abatacept, apremilast, secukinumab and ustekinumab treatment of psoriatic arthritis: a systematic review and network meta-analysis
Source: Rheumatol Int. 2017 Dec 28;38(2):189–201. doi: 10.1007/s00296-017-3919-7 (PMC5773655; doi:10.1007/s00296-017-3919-7)
Supplement: Supplementary file 1 — Supplementary material 1 (DOCX 81 KB) [file 296_2017_3919_MOESM1_ESM.docx]

**SUPLEMANTARY MATERIAL 1**

**Table S1. Analysis of homogeneity of included studies.**

| **Reference** | **Methodology** | **Population** | **Inclusion criteria** | **Treatment regimen** | **Concomitant therapy** |
| --- | --- | --- | --- | --- | --- |
| **Abatacept vs placebo** | | | | | |
| **Mease 2011 [29]** | RCT, phase 2, multicenter, double-blind, multiple dose-level, placebo controlled | Active PsA despite DMARDs, or anti-TNF agents | 1. At least 18-year old  2. Fulfilled the classification criteria for psoriatic arthritis (CASPAR)  3. Active PsA (i.e., ≥3 swollen joints and ≥3 tender joints  4. History of an inadequate response or intolerance to DMARDs (including, but not limited to MTX) or anti-TNF agents | 1. Abatacept 3 mg/kg (n=45), 10 mg/kg (n=40) or 30/10 mg/kg (2 initial doses of 30 mg/kg, followed by 10 mg/kg, n=43) on days 1, 15, and 29 and then once every 28 days thereafter for 24 weeks  2. Placebo (n=42) | Methotrexate was continued at a stable dosage only if it had been taken at a stable dosage for ≥3 months prior to screening. Other DMARDs were discontinued.  Corticosteroids were allowed if the dosage (≤10 mg of prednisone or its equivalent) had been stable for ≥28 days. Stable regimens of NSAIDs were allowed throughout the study. |
| **Apremilast vs placebo** | | | | | |
| **PALACE 1**  **Kavanaugh 2014 [30]** | RCT, phase 3, multicentre (83 sites in 13 countries), double-blind, placebo controlled | Active PsA despite DMARDs or/and biologic agents (anti-TNF, tocilizumab, alefacept, ustekinumab) | 1. At least 18-year old  2. Fulfilled the classification criteria for psoriatic arthritis (CASPAR)  3. Active PsA (i.e., ≥ 3 swollen joints and ≥ 3 tender joints)  4. History of an inadequate response or intolerance to DMARDs or biologic agents | 1. Apremilast 20 mg twice daily (n=168) or apremilast 30 mg twice daily (n=168) for 24 weeks  2. Placebo (n=168) | Methotrexate, leflunomide or sulfasalazine were allowed if patients had been treated for ≥16 weeks and on a stable dose (MTX ≤25 mg/wk; leflunomide ≤20 mg/d; sulfasalazine ≤2 g/d) for ≥4 weeks before the screening visit. Stable doses of oral corticosteroids (prednisone ≤10mg/day or equivalent) and NSAIDs (≥2 weeks) were also permitted |
| **PALACE 2**  **Cutolo 2016 [31]** | RCT, phase 3, multicentre, randomized, double-blind, placebo controlled | Active PsA despite DMARDs or/and biologic agents  (anti-TNF, tocilizumab, alefacept, ustekinumab) | 1. At least 18-year old  2. Fulfilled the classification criteria for psoriatic arthritis (CASPAR)  3. Active PsA (i.e., ≥ 3 swollen joints and ≥ 3 tender joints)  4. History of an inadequate response or intolerance to DMARDs or biologic agents | 1. Apremilast 20 mg twice daily (n=163) or apremilast 30 mg twice daily (n=162) for 24 weeks  2. Placebo (n=159) | Methotrexate, leflunomide or sulfasalazine were allowed if patients had been treated for ≥16 weeks and on a stable dose (MTX ≤25 mg/wk; leflunomide ≤20 mg/d; sulfasalazine ≤2 g/d) for at least 4 weeks before the screening visit. Stable doses of corticosteroids (prednisone ≤10mg/day or equivalent) and NSAIDs (≥2 weeks) were also permitted |
| **PALACE 3**  **Edwards 2013 [32]** | RCT, phase 3, multicentre, double-blind, placebo controlled | Active PsA despite DMARDs or/and biologic agents | 1. Active PsA  2. At least 1 psoriatic lesion ≥2 cm  3. History of an inadequate response or intolerance to DMARDs or biologic agents | 1. Apremilast 20 mg twice daily (n=169) or apremilast 30 mg twice daily (n=167) for 24 weeks  2. Placebo (n=169) | Methotrexate, leflunomide, sulfasalazine (or combination) were allowed to continue at a stable dose throughout the study |
| **Secukinumab vs placebo** | | | | | |
| **FUTURE 1**  **Mease 2015 [33]** | RCT, phase 3, multicentre (104 sites in North America and South America, Europe, the Middle East, Australia and Asia), double-blind, placebo controlled | Active PsA despite NSAIDs, DMARDs, or anti-TNF agents | 1. At least 18-year old  2. Fulfilled the classification criteria for psoriatic arthritis (CASPAR)  3. Active PsA (i.e., ≥3 swollen joints and ≥3 tender joints  4. History of an inadequate response or intolerance to DMARDs, NSAIDs or anti-TNF therapy | 1. Secukinumab 10 mg/kg at baseline, week 2, and week 4 then 75 mg (n=202) or 150 mg (n=202) subcutaneously every 4 weeks  2. Placebo (n=202) | The concomitant use of methotrexate (at a dose of ≤25 mg per week) and oral glucocorticoids (≤10 mg/ day of prednisone or its equivalent) was permitted, provided that the dose was stable |
| **FUTURE 2**  **McInnes 2015 [34]** | RCT, phase 3, multicentre (76 sites in Asia, Australia, Canada, Europe, and the USA), double-blind, placebo controlled | Active PsA despite NSAIDs, DMARDs or anti-TNF agents | 1. At least 18-year old  2. Fulfilled the classification criteria for psoriatic arthritis (CASPAR)  3. Active PsA (i.e., ≥3 swollen joints and ≥3 tender joints  4. History of an inadequate response or intolerance to DMARDs, NSAIDs or anti-TNF therapy | 1. Secukinumab 75 mg (n=99), 150 mg (n=100) and 300 mg (n=100) weekly for 4 weeks then every 4 weeks  2. Placebo (n=98) | Methotrexate was allowed to continue if the dose was stable for 4 weeks before study start (≤25 mg/ wk). Other DMARDs were discontinued. Corticosteroids were allowed if the dose was ≤10 mg/ day of prednisone |
| **Ustekinumab vs placebo** | | | | | |
| **PSUMMIT 1**  **McInnes 2013 [35]** | RCT, phase 3, multicenter (104 sites in 14 countries), double-blind, placebo controlled | Active PsA despite DMARDs or/and NSAIDs | 1. At least 18-year old  2. Active PsA (i.e., ≥5 swollen joints and ≥5 tender joints, CRP ≥3.0 mg/L)  3. Active psoriatic skin lesion or a documented history of psoriasis  4. History of an inadequate response or intolerance to DMARDs or NSAIDs  5. Never took anti-TNF therapy | 1. Ustekinumab 45 mg (n=205) and 90 mg (n=204) subcutaneously at baseline, week 4 and then every 12 weeks  2. Placebo (n=206) | Methotrexate was allowed if started ≥3 months prior to the start of study agent and if taken at a stable dose (≤25 mg/wk) for ≥4 weeks. Stable regimens (≥2 weeks) of NSAIDs and/ or oral corticosteroids (≤10 mg prednisone/ day) were also allowed |
| **PSUMMIT 2**  **Ritchlin 2014 [36]** | RCT, phase 3, multicentre, double-blind, placebo controlled | Active PsA despite DMARDs, NSAIDs or/and anti-TNF agents | 1. At least 18-year old  2. Active PsA (i.e., ≥5 swollen joints and ≥5 tender joints, CRP ≥3.0 mg/L)  3. Active psoriatic skin lesion or a documented history of psoriasis  4. History of an inadequate response or intolerance to DMARDs or NSAIDs or anti-TNF therapy | 1. Ustekinumab 45 mg (n=103) and 90 mg (n=105) subcutaneously at baseline, week 4 and then every 12 weeks  2. Placebo (n=104) | Methotrexate was allowed if started ≥3 months prior to the start of study agent and if taken at a stable dose (≤25 mg/wk) for ≥4 weeks. Stable regimens (≥2 weeks) of NSAIDs and/ or oral corticosteroids (≤10 mg prednisone/ day) were also allowed |

PsA – Psoriatic arthritis, RCT – Randomized clinical trial

**Table S2. P-Score (overall rank based on P-Score).**

|  | A1. ACR20, overall population | A2. ACR50, overall population | A3. PASI75, overall population | A4. Any adverse event | A5. Severe adverse events | A6. Withdrawal due to adverse events | A7. ACR20, anti-TNF naïve patients | A8. ACR20, anti-TNF failure patients | A9. ACR20, anti-TNF experienced patients |
| --- | --- | --- | --- | --- | --- | --- | --- | --- | --- |
| ABA_03 | 0.313 (9) | 0.620 (5) | 0.670 (4) | 0.706 (3) | 0.762 (2) | 0.708 (3) | 0.300 (9) | 0.411 (8) | 0.428 (8) |
| ABA_10 | 0.639 (3) | 0.829 (1) | 0.323 (8) | 0.370 (9) | 0.320 (9) | 0.462 (6) | 0.712 (3) | 0.405 (9) | 0.421 (9) |
| ABA_30/10 | 0.512 (6) | 0.751 (3) | 0.235 (10) | 0.752 (2) | 0.151 (11) | 0.696 (4) | 0.561 (5) | 0.509 (6) | 0.529 (6) |
| APR_20 | 0.251 (10) | 0.248 (10) | 0.321 (9) | 0.112 (11) | 0.531 (6) | 0.204 (10) | 0.200 (10) | 0.669 (2) | 0.456 (7) |
| APR_30 | 0.407 (8) | 0.291 (9) | 0.386 (7) | 0.123 (10) | 0.489 (7) | 0.076 (11) | 0.354 (8) | 0.608 (3) | 0.630 (2) |
| PLC | 0.015 (11) | 0.009 (11) | 0.049 (11) | 0.701 (4) | 0.468 (8) | 0.325 (9) | 0.016 (11) | 0.064 (11) | 0.057 (11) |
| SEC_075 | 0.595 (4) | 0.482 (7) | 0.491 (6) | 0.771 (1) | 0.577 (4) | 0.434 (7) | 0.685 (4) | 0.367 (10) | 0.381 (10) |
| SEC_150 | 0.815 (2) | 0.706 (4) | 0.604 (5) | 0.473 (7) | 0.561 (5) | 0.629 (5) | 0.867 (2) | 0.508 (7) | 0.536 (5) |
| SEC_300 | 0.932 (1) | 0.759 (2) | 0.794 (2) | 0.567 (5) | 0.273 (10) | 0.420 (8) | 0.899 (1) | 0.848 (1) | 0.888 (1) |
| UST_45 | 0.510 (7) | 0.324 (8) | 0.789 (3) | 0.410 (8) | 0.594 (3) | 0.794 (1) | 0.380 (7) | 0.583 (4) | 0.616 (3) |
| UST_90 | 0.513 (5) | 0.483 (6) | 0.838 (1) | 0.517 (6) | 0.775 (1) | 0.752 (2) | 0.526 (6) | 0.529 (5) | 0.558 (4) |

**Table S3. Results of network meta-analyses: odds ratios with 95% CIs for all comparisons. OR below treatment labels >1 means that the bottom right treatment increases the odds for an outcome; OR above treatment labels >1 means that the top left treatment increases the odds for an outcome (efficacy outcomes: the treatment is better; safety outcomes: the treatment is worse).**

| **A1. ACR20, overall population** | | | | | | | | | | |
| --- | --- | --- | --- | --- | --- | --- | --- | --- | --- | --- |
| ABA_03 | 0.55 (0.19, 1.58) | 0.69 (0.25, 1.97) | 1.04 (0.30, 3.54) | 0.82 (0.24, 2.81) | 2.13 (0.68, 6.67) | 0.59 (0.16, 2.12) | 0.41 (0.11, 1.46) | 0.29 (0.07, 1.15) | 0.69 (0.17, 2.87) | 0.69 (0.17, 2.86) |
| 1.81 (0.63, 5.17) | ABA_10 | 1.26 (0.44, 3.56) | 1.88 (0.55, 6.41) | 1.49 (0.44, 5.09) | **3.85 (1.22, 12.08)** | 1.07 (0.30, 3.84) | 0.74 (0.21, 2.64) | 0.53 (0.13, 2.08) | 1.25 (0.30, 5.20) | 1.25 (0.30, 5.17) |
| 1.44 (0.51, 4.08) | 0.80 (0.28, 2.25) | ABA_30/10 | 1.49 (0.44, 5.07) | 1.19 (0.35, 4.02) | 3.06 (0.98, 9.54) | 0.85 (0.24, 3.04) | 0.59 (0.16, 2.09) | 0.42 (0.11, 1.64) | 1.00 (0.24, 4.11) | 0.99 (0.24, 4.09) |
| 0.96 (0.28, 3.29) | 0.53 (0.16, 1.82) | 0.67 (0.20; 2.27) | APR_20 | 0.79 (0.52, 1.21) | **2.05 (1.31, 3.19)** | 0.57 (0.28, 1.17) | **0.39 (0.19, 0.81)** | **0.28 (0.12, 0.68)** | 0.67 (0.26, 1.73) | 0.66 (0.26, 1.72) |
| 1.21 (0.36, 4.13) | 0.67 (0.20, 2.29) | 0.84 (0.25, 2.85) | 1.26 (0.82, 1.92) | APR_30 | **2.58 (1.66, 4.01)** | 0.72 (0.35, 1.48) | 0.49 (0.24, 1.01) | **0.35 (0.15, 0.85)** | 0.84 (0.32, 2.18) | 0.84 (0.32, 2.16) |
| 0.47 (0.15, 1.48) | **0.26 (0.08, 0.82)** | 0.33 (0.10, 1.02) | **0.49 (0.31, 0.76)** | **0.39 (0.25, 0.60)** | PLC | **0.28 (0.16, 0.49)** | **0.19 (0.11, 0.34)** | **0.14 (0.06, 0.29)** | **0.33 (0.14, 0.76)** | **0.32 (0.14, 0.75)** |
| 1.69 (0.47, 6.08) | 0.94 (0.26, 3.36) | 1.18 (0.33, 4.19) | 1.76 (0.85, 3.62) | 1.40 (0.68, 2.87) | **3.60 (2.03, 6.36)** | SEC_075 | 0.69 (0.41, 1.17) | 0.49 (0.24, 1.02) | 1.17 (0.42, 3.25) | 1.17 (0.42, 3.23) |
| 2.45 (0.68, 8.80) | 1.36 (0.38, 4.86) | 1.70 (0.48, 6.07) | **2.55 (1.24, 5.23)** | 2.02 (0.99, 4.15) | **5.22 (2.96, 9.19)** | 1.45 (0.85, 2.47) | SEC_150 | 0.71 (0.35, 1.47) | 1.70 (0.62, 4.70) | 1.69 (0.61, 4.67) |
| 3.44 (0.87, 13.62) | 1.90 (0.48, 7.53) | 2.39 (0.61, 9.40) | **3.57 (1.48, 8.64)** | **2.84 (1.18, 6.86)** | **7.32 (3.41, 15.68)** | 2.04 (0.98, 4.24) | 1.40 (0.68, 2.90) | SEC_300 | 2.39 (0.77, 7.44) | 2.37 (0.76, 7.40) |
| 1.44 (0.35, 5.98) | 0.80 (0.19, 3.31) | 1.00 (0.24, 4.13) | 1.50 (0.58, 3.89) | 1.19 (0.46, 3.09) | **3.07 (1.32, 7.13)** | 0.85 (0.31, 2.36) | 0.59 (0.21, 1.62) | 0.42 (0.13, 1.31) | UST_45 | 1.00 (0.45, 2.20) |
| 1.45 (0.35, 6.00) | 0.80 (0.19, 3.32) | 1.01 (0.24, 4.14) | 1.51 (0.58, 3.90) | 1.20 (0.46, 3.10) | **3.08 (1.33, 7.16)** | 0.86 (0.31, 2.37) | 0.59 (0.21, 1.63) | 0.42 (0.14, 1.31) | 1.00 (0.45, 2.23) | UST_90 |
| **A2. ACR50, overall population** | | | | | | | | | | |
| ABA_03 | 0.55 (0.17, 1.85) | 0.70 (0.21, 2.35) | 2.96 (0.30, 28.89) | 2.74 (0.28, 26.73) | 7.55 (0.83, 68.73) | 1.73 (0.17, 17.31) | 1.10 (0.11, 10.96) | 0.94 (0.09, 9.95) | 2.57 (0.22, 29.90) | 1.84 (0.16, 21.15) |
| 1.81 (0.54, 6.04) | ABA_10 | 1.26 (0.40, 4.01) | 5.36 (0.57, 50.70) | 4.96 (0.53, 46.92) | **13.67 (1.55, 120.53)** | 3.14 (0.32, 30.39) | 1.99 (0.21, 19.25) | 1.71 (0.17, 17.49) | 4.66 (0.41, 52.61) | 3.33 (0.30, 37.20) |
| 1.44 (0.43, 4.85) | 0.79 (0.25, 2.53) | ABA_30/10 | 4.26 (0.45, 40.51) | 3.94 (0.41, 37.49) | **10.85 (1.22, 96.32)** | 2.49 (0.26, 24.28) | 1.58 (0.16, 15.38) | 1.35 (0.13, 13.97) | 3.70 (0.33, 42.01) | 2.64 (0.24, 29.71) |
| 0.34 (0.03, 3.29) | 0.19 (0.02, 1.76) | 0.23 (0.02; 2.24) | APR_20 | 0.93 (0.58, 1.49) | **2.55 (1.46, 4.45)** | 0.59 (0.25, 1.37) | **0.37 (0.16, 0.86)** | **0.32 (0.12, 0.86)** | 0.87 (0.26, 2.89) | 0.62 (0.19, 2.02) |
| 0.36 (0.04, 3.55) | 0.20 (0.02, 1.90) | 0.25 (0.03, 2.41) | 1.08 (0.67, 1.74) | APR_30 | **2.75 (1.58, 4.79)** | 0.63 (0.27, 1.48) | **0.40 (0.17, 0.93)** | **0.34 (0.13, 0.93)** | 0.94 (0.28, 3.12) | 0.67 (0.21, 2.19) |
| 0.13 (0.01, 1.21) | **0.07 (0.01, 0.65)** | **0.09 (0.01, 0.82)** | **0.39 (0.23, 0.68)** | **0.36 (0.21, 0.63)** | PLC | **0.23 (0.12, 0.44)** | **0.15 (0.08, 0.28)** | **0.12 (0.05, 0.28)** | **0.34 (0.12, 0.99)** | **0.24 (0.09, 0.69)** |
| 0.58 (0.06, 5.76) | 0.32 (0.03, 3.09) | 0.40 (0.04, 3.91) | 1.71 (0.73, 4.01) | 1.58 (0.68, 3.71) | **4.36 (2.28, 8.31)** | SEC_075 | 0.64 (0.37, 1.08) | 0.54 (0.26, 1.15) | 1.48 (0.43, 5.17) | 1.06 (0.31, 3.62) |
| 0.91 (0.09, 9.03) | 0.50 (0.05, 4.84) | 0.63 (0.07, 6.14) | **2.69 (1.16, 6.25)** | **2.49 (1.07, 5.78)** | **6.86 (3.64, 12.93)** | 1.57 (0.92, 2.68) | SEC_150 | 0.86 (0.41, 1.77) | 2.34 (0.68, 8.08) | 1.67 (0.49, 5.66) |
| 1.06 (0.10, 11.20) | 0.59 (0.06, 6.01) | 0.74 (0.07, 7.61) | **3.14 (1.16, 8.49)** | **2.91 (1.08, 7.85)** | **8.01 (3.52, 18.25)** | 1.84 (0.87, 3.88) | 1.17 (0.57, 2.41) | SEC_300 | 2.73 (0.71, 10.51) | 1.95 (0.52, 7.37) |
| 0.39 (0.03, 4.51) | 0.21 (0.02, 2.43) | 0.27 (0.02, 3.07) | 1.15 (0.35, 3.83) | 1.07 (0.32, 3.55) | **2.93 (1.01, 8.53)** | 0.67 (0.19, 2.35) | 0.43 (0.12, 1.48) | 0.37 (0.10, 1.41) | UST_45 | 0.71 (0.30, 1.71) |
| 0.54 (0.05, 6.25) | 0.30 (0.03, 3.36) | 0.38 (0.03, 4.25) | 1.61 (0.49, 5.25) | 1.49 (0.46, 4.86) | **4.11 (1.45, 11.65)** | 0.94 (0.28, 3.21) | 0.60 (0.18, 2.03) | 0.51 (0.14, 1.94) | 1.40 (0.59, 3.34) | UST_90 |
| **A3. PASI75, overall population with significant body surface area affected by psoriasis** | | | | | | | | | | |
| ABA_03 | 3.69 (0.58, 23.36) | 5.54 (0.74, 41.38) | 2.93 (0.22, 39.77) | 2.47 (0.18, 33.54) | **12.31 (1.08, 140.92)** | 1.70 (0.12, 23.58) | 1.27 (0.09, 17.57) | 0.68 (0.04, 10.73) | 0.62 (0.03, 10.95) | 0.52 (0.03, 9.21) |
| 0.27 (0.04, 1.71) | ABA_10 | 1.50 (0.17, 13.30) | 0.79 (0.05, 12.32) | 0.67 (0.04, 10.39) | 3.33 (0.25, 44.04) | 0.46 (0.03, 7.29) | 0.34 (0.02, 5.44) | 0.18 (0.01, 3.30) | 0.17 (0.01, 3.35) | 0.14 (0.01, 2.82) |
| 0.18 (0.02, 1.35) | 0.67 (0.08, 5.91) | ABA_30/10 | 0.53 (0.03, 9.21) | 0.45 (0.03, 7.77) | 2.22 (0.15, 33.14) | 0.31 (0.02, 5.45) | 0.23 (0.01, 4.06) | 0.12 (0.01, 2.45) | 0.11 (0.01, 2.48) | 0.09 (0.00, 2.09) |
| 0.34 (0.03, 4.65) | 1.26 (0.08, 19.62) | 1.89 (0.11; 32.99) | APR_20 | 0.85 (0.40, 1.80) | **4.21 (1.66, 10.67)** | 0.58 (0.15, 2.25) | 0.44 (0.11, 1.67) | 0.23 (0.05, 1.15) | 0.21 (0.04, 1.26) | 0.18 (0.03, 1.06) |
| 0.40 (0.03, 5.49) | 1.49 (0.10, 23.16) | 2.24 (0.13, 38.96) | 1.18 (0.55, 2.52) | APR_30 | **4.98 (1.97, 12.55)** | 0.69 (0.18, 2.66) | 0.51 (0.13, 1.97) | 0.27 (0.06, 1.35) | 0.25 (0.04, 1.49) | 0.21 (0.04, 1.25) |
| **0.08 (0.01, 0.93)** | 0.30 (0.02, 3.96) | 0.45 (0.03, 6.71) | **0.24 (0.09, 0.60)** | **0.20 (0.08, 0.51)** | PLC | **0.14 (0.05, 0.37)** | **0.10 (0.04, 0.27)** | **0.05 (0.01, 0.20)** | **0.05 (0.01, 0.23)** | **0.04 (0.01, 0.19)** |
| 0.59 (0.04, 8.16) | 2.17 (0.14, 34.40) | 3.26 (0.18, 57.81) | 1.72 (0.44, 6.67) | 1.45 (0.38, 5.62) | **7.24 (2.70, 19.39)** | SEC_075 | 0.75 (0.31, 1.83) | 0.40 (0.11, 1.39) | 0.36 (0.06, 2.23) | 0.30 (0.05, 1.88) |
| 0.79 (0.06, 10.84) | 2.90 (0.18, 45.74) | 4.35 (0.25, 76.87) | 2.30 (0.60, 8.83) | 1.94 (0.51, 7.43) | **9.67 (3.66, 25.56)** | 1.34 (0.55, 3.26) | SEC_150 | 0.53 (0.15, 1.83) | 0.48 (0.08, 2.96) | 0.41 (0.07, 2.49) |
| 1.48 (0.09, 23.52) | 5.47 (0.30, 98.61) | 8.20 (0.41, 164.90) | 4.33 (0.87, 21.53) | 3.66 (0.74, 18.13) | **18.22 (4.94, 67.25)** | 2.52 (0.72, 8.82) | 1.88 (0.55, 6.49) | SEC_300 | 0.91 (0.12, 6.81) | 0.77 (0.10, 5.73) |
| 1.62 (0.09, 28.84) | 5.99 (0.30, 120.34) | 8.99 (0.40, 200.48) | 4.75 (0.79, 28.43) | 4.01 (0.67, 23.96) | **19.97 (4.33, 92.14)** | 2.76 (0.45, 17.01) | 2.07 (0.34, 12.65) | 1.10 (0.15, 8.19) | UST_45 | 0.84 (0.25, 2.88) |
| 1.93 (0.11, 34.30) | 7.13 (0.35, 143.09) | 10.69 (0.48, 238.37) | 5.65 (0.94, 33.81) | 4.77 (0.80, 28.49) | **23.75 (5.15, 109.55)** | 3.28 (0.53, 20.22) | 2.46 (0.40, 15.04) | 1.30 (0.17, 9.73) | 1.19 (0.35, 4.08) | UST_90 |
| **A4. Any adverse event, overall population** | | | | | | | | | | |
| ABA_03 | 0.64 (0.24, 1.70) | 1.07 (0.44, 2.62) | 0.51 (0.20, 1.33) | 0.52 (0.20, 1.34) | 0.89 (0.35, 2.22) | 0.95 (0.36, 2.53) | 0.75 (0.28, 1.99) | 0.81 (0.28, 2.30) | 0.71 (0.27, 1.87) | 0.77 (0.29, 2.04) |
| 1.56 (0.59, 4.12) | ABA_10 | 1.66 (0.63, 4.42) | 0.79 (0.28, 2.23) | 0.80 (0.29, 2.25) | 1.38 (0.51, 3.74) | 1.48 (0.52, 4.25) | 1.17 (0.41, 3.34) | 1.26 (0.41, 3.84) | 1.10 (0.38, 3.14) | 1.20 (0.42, 3.42) |
| 0.94 (0.38, 2.29) | 0.60 (0.23, 1.60) | ABA_30/10 | 0.48 (0.18, 1.25) | 0.48 (0.19, 1.26) | 0.83 (0.33, 2.09) | 0.89 (0.34, 2.38) | 0.70 (0.26, 1.87) | 0.76 (0.27, 2.16) | 0.66 (0.25, 1.76) | 0.72 (0.27, 1.91) |
| 1.96 (0.75, 5.08) | 1.26 (0.45, 3.53) | 2.09 (0.80; 5.46) | APR_20 | 1.01 (0.78, 1.31) | **1.73 (1.35, 2.23)** | **1.87 (1.24, 2.82)** | 1.47 (0.97, 2.22) | 1.58 (0.91, 2.76) | 1.38 (0.92, 2.08) | 1.50 (1.00, 2.26) |
| 1.93 (0.75, 5.02) | 1.24 (0.44, 3.49) | 2.07 (0.79, 5.39) | 0.99 (0.77, 1.28) | APR_30 | **1.71 (1.33, 2.21)** | **1.85 (1.22, 2.79)** | 1.45 (0.96, 2.19) | 1.57 (0.90, 2.73) | 1.37 (0.91, 2.06) | 1.49 (0.99, 2.24) |
| 1.13 (0.45, 2.83) | 0.73 (0.27, 1.97) | 1.21 (0.48, 3.04) | **0.58 (0.45, 0.74)** | **0.58 (0.45, 0.75)** | PLC | 1.08 (0.78, 1.49) | 0.85 (0.61, 1.17) | 0.91 (0.56, 1.50) | 0.80 (0.58, 1.10) | 0.87 (0.63, 1.20) |
| 1.05 (0.40, 2.78) | 0.67 (0.24, 1.93) | 1.12 (0.42, 2.98) | **0.54 (0.35, 0.81)** | **0.54 (0.36, 0.82)** | 0.93 (0.67, 1.28) | SEC_075 | 0.79 (0.57, 1.09) | 0.85 (0.52, 1.39) | 0.74 (0.47, 1.17) | 0.81 (0.51, 1.27) |
| 1.33 (0.50, 3.54) | 0.86 (0.30, 2.45) | 1.43 (0.53, 3.80) | 0.68 (0.45, 1.03) | 0.69 (0.46, 1.04) | 1.18 (0.85, 1.64) | 1.27 (0.92, 1.77) | SEC_150 | 1.08 (0.66, 1.77) | 0.94 (0.60, 1.49) | 1.03 (0.65, 1.62) |
| 1.24 (0.43, 3.51) | 0.79 (0.26, 2.42) | 1.32 (0.46, 3.77) | 0.63 (0.36, 1.10) | 0.64 (0.37, 1.11) | 1.09 (0.67, 1.79) | 1.18 (0.72, 1.93) | 0.93 (0.57, 1.52) | SEC_300 | 0.87 (0.48, 1.58) | 0.95 (0.53, 1.71) |
| 1.42 (0.53, 3.75) | 0.91 (0.32, 2.60) | 1.51 (0.57, 4.03) | 0.72 (0.48, 1.09) | 0.73 (0.49, 1.10) | 1.25 (0.91, 1.73) | 1.35 (0.85, 2.14) | 1.06 (0.67, 1.68) | 1.15 (0.63, 2.07) | UST_45 | 1.09 (0.79, 1.51) |
| 1.30 (0.49, 3.45) | 0.84 (0.29, 2.39) | 1.39 (0.52, 3.70) | 0.66 (0.44, 1.00) | 0.67 (0.45, 1.01) | 1.15 (0.84, 1.59) | 1.24 (0.79, 1.96) | 0.98 (0.62, 1.54) | 1.05 (0.58, 1.90) | 0.92 (0.66, 1.27) | UST_90 |
| **A5. Severe adverse events, overall population** | | | | | | | | | | |
| ABA_03 | 0.17 (0.01, 3.95) | 0.10 (0.00, 2.01) | 0.33 (0.01, 10.08) | 0.31 (0.01, 9.33) | 0.30 (0.01, 8.30) | 0.37 (0.01, 11.80) | 0.36 (0.01, 11.47) | 0.18 (0.00, 6.60) | 0.39 (0.01, 13.42) | 0.60 (0.02, 19.94) |
| 5.91 (0.25, 137.82) | ABA_10 | 0.57 (0.10, 3.32) | 1.98 (0.19, 20.48) | 1.84 (0.18, 18.90) | 1.80 (0.20, 16.09) | 2.16 (0.19, 24.71) | 2.10 (0.18, 23.99) | 1.04 (0.07, 14.65) | 2.33 (0.19, 28.77) | 3.54 (0.30, 42.31) |
| 10.37 (0.50, 216.44) | 1.75 (0.30, 10.21) | ABA_30/10 | 3.47 (0.39, 30.89) | 3.23 (0.37, 28.48) | 3.15 (0.41, 23.99) | 3.79 (0.38, 37.50) | 3.69 (0.37, 36.41) | 1.83 (0.15, 22.49) | 4.08 (0.38, 43.87) | 6.21 (0.60, 64.40) |
| 2.99 (0.10, 90.16) | 0.51 (0.05, 5.25) | 0.29 (0.03; 2.57) | APR_20 | 0.93 (0.42, 2.08) | 0.91 (0.40, 2.05) | 1.09 (0.29, 4.18) | 1.06 (0.28, 4.05) | 0.53 (0.10, 2.85) | 1.18 (0.27, 5.16) | 1.79 (0.43, 7.41) |
| 3.21 (0.11, 95.99) | 0.54 (0.05, 5.57) | 0.31 (0.04, 2.73) | 1.07 (0.48, 2.39) | APR_30 | 0.98 (0.45, 2.14) | 1.17 (0.31, 4.40) | 1.14 (0.31, 4.26) | 0.57 (0.11, 3.01) | 1.26 (0.29, 5.44) | 1.92 (0.47, 7.81) |
| 3.29 (0.12, 89.80) | 0.56 (0.06, 4.98) | 0.32 (0.04, 2.42) | 1.10 (0.49, 2.48) | 1.03 (0.47, 2.25) | PLC | 1.20 (0.42, 3.48) | 1.17 (0.41, 3.37) | 0.58 (0.13, 2.54) | 1.30 (0.38, 4.44) | 1.97 (0.61, 6.30) |
| 2.73 (0.08, 88.22) | 0.46 (0.04, 5.29) | 0.26 (0.03, 2.61) | 0.91 (0.24, 3.49) | 0.85 (0.23, 3.20) | 0.83 (0.29, 2.41) | SEC_075 | 0.97 (0.32, 3.00) | 0.48 (0.12, 1.94) | 1.08 (0.21, 5.48) | 1.64 (0.34, 7.92) |
| 2.81 (0.09, 90.45) | 0.48 (0.04, 5.42) | 0.27 (0.03, 2.67) | 0.94 (0.25, 3.57) | 0.88 (0.23, 3.27) | 0.85 (0.30, 2.46) | 1.03 (0.33, 3.17) | SEC_150 | 0.50 (0.11, 2.34) | 1.11 (0.22, 5.61) | 1.68 (0.35, 8.10) |
| 5.67 (0.15, 211.86) | 0.96 (0.07, 13.47) | 0.55 (0.04, 6.72) | 1.89 (0.35, 10.22) | 1.77 (0.33, 9.40) | 1.72 (0.39, 7.53) | 2.07 (0.51, 8.34) | 2.02 (0.43, 9.52) | SEC_300 | 2.23 (0.33, 15.26) | 3.39 (0.52, 22.21) |
| 2.54 (0.07, 86.60) | 0.43 (0.03, 5.31) | 0.25 (0.02, 2.63) | 0.85 (0.19, 3.72) | 0.79 (0.18, 3.41) | 0.77 (0.23, 2.65) | 0.93 (0.18, 4.73) | 0.90 (0.18, 4.59) | 0.45 (0.07, 3.06) | UST_45 | 1.52 (0.40, 5.83) |
| 1.67 (0.05, 55.65) | 0.28 (0.02, 3.38) | 0.16 (0.02, 1.67) | 0.56 (0.13, 2.31) | 0.52 (0.13, 2.12) | 0.51 (0.16, 1.63) | 0.61 (0.13, 2.96) | 0.59 (0.12, 2.87) | 0.29 (0.05, 1.93) | 0.66 (0.17, 2.52) | UST_90 |
| **A6. Withdrawal due to adverse events, overall population** | | | | | | | | | | |
| ABA_03 | 0.43 (0.04, 4.95) | 0.95 (0.06, 15.76) | 0.22 (0.02, 2.42) | 0.17 (0.02, 1.81) | 0.30 (0.03, 2.96) | 0.39 (0.03, 4.90) | 0.65 (0.05, 9.09) | 0.38 (0.02, 6.03) | 1.07 (0.09, 13.29) | 0.93 (0.08, 11.22) |
| 2.32 (0.20, 26.55) | ABA_10 | 2.21 (0.19, 25.37) | 0.52 (0.08, 3.59) | 0.39 (0.06, 2.69) | 0.68 (0.11, 4.33) | 0.91 (0.11, 7.52) | 1.51 (0.16, 14.24) | 0.87 (0.08, 9.67) | 2.48 (0.30, 20.38) | 2.15 (0.27, 17.10) |
| 1.05 (0.06, 17.29) | 0.45 (0.04, 5.19) | ABA_30/10 | 0.24 (0.02, 2.53) | 0.18 (0.02, 1.90) | 0.31 (0.03, 3.10) | 0.41 (0.03, 5.14) | 0.68 (0.05, 9.53) | 0.39 (0.02, 6.33) | 1.12 (0.09, 13.94) | 0.97 (0.08, 11.76) |
| 4.46 (0.41, 47.97) | 1.92 (0.28, 13.31) | 4.25 (0.39; 45.83) | APR_20 | 0.75 (0.45, 1.26) | 1.32 (0.74, 2.36) | 1.75 (0.53, 5.73) | 2.90 (0.71, 11.86) | 1.68 (0.32, 8.74) | **4.77 (1.48, 15.41)** | **4.14 (1.37, 12.57)** |
| 5.93 (0.55, 63.50) | 2.56 (0.37, 17.60) | 5.66 (0.53, 60.67) | 1.33 (0.79, 2.23) | APR_30 | 1.75 (1.00, 3.08) | 2.32 (0.72, 7.55) | 3.85 (0.95, 15.65) | 2.23 (0.43, 11.55) | **6.35 (1.98, 20.30)** | **5.51 (1.84, 16.55)** |
| 3.38 (0.34, 33.89) | 1.46 (0.23, 9.24) | 3.23 (0.32, 32.38) | 0.76 (0.42, 1.36) | 0.57 (0.33, 1.00) | PLC | 1.33 (0.47, 3.73) | 2.20 (0.61, 7.94) | 1.28 (0.27, 5.97) | **3.63 (1.31, 10.02)** | **3.15 (1.22, 8.09)** |
| 2.55 (0.20, 31.87) | 1.10 (0.13, 9.12) | 2.43 (0.19, 30.45) | 0.57 (0.17, 1.88) | 0.43 (0.13, 1.40) | 0.75 (0.27, 2.12) | SEC_075 | 1.66 (0.44, 6.28) | 0.96 (0.19, 4.75) | 2.73 (0.64, 11.65) | 2.37 (0.58, 9.63) |
| 1.54 (0.11, 21.48) | 0.66 (0.07, 6.28) | 1.47 (0.10, 20.52) | 0.35 (0.08, 1.41) | 0.26 (0.06, 1.05) | 0.45 (0.13, 1.64) | 0.60 (0.16, 2.28) | SEC_150 | 0.58 (0.09, 3.67) | 1.65 (0.32, 8.47) | 1.43 (0.29, 7.04) |
| 2.65 (0.17, 42.47) | 1.15 (0.10, 12.69) | 2.53 (0.16, 40.57) | 0.60 (0.11, 3.10) | 0.45 (0.09, 2.32) | 0.78 (0.17, 3.67) | 1.04 (0.21, 5.14) | 1.73 (0.27, 10.92) | SEC_300 | 2.84 (0.45, 18.05) | 2.47 (0.40, 15.07) |
| 0.93 (0.08, 11.58) | 0.40 (0.05, 3.31) | 0.89 (0.07, 11.07) | **0.21 (0.06, 0.68)** | **0.16 (0.05, 0.50)** | **0.28 (0.10, 0.76)** | 0.37 (0.09, 1.56) | 0.61 (0.12, 3.12) | 0.35 (0.06, 2.23) | UST_45 | 0.87 (0.26, 2.88) |
| 1.08 (0.09, 12.96) | 0.46 (0.06, 3.69) | 1.03 (0.09, 12.38) | **0.24 (0.08, 0.73)** | **0.18 (0.06, 0.54)** | **0.32 (0.12, 0.82)** | 0.42 (0.10, 1.71) | 0.70 (0.14, 3.44) | 0.41 (0.07, 2.47) | 1.15 (0.35, 3.83) | UST_90 |
| **A7. ACR20, anti-TNF naïve patient population** | | | | | | | | | | |
| ABA_03 | 0.42 (0.13, 1.35) | 0.58 (0.17, 1.98) | 1.13 (0.30, 4.24) | 0.87 (0.23, 3.28) | 2.11 (0.60, 7.41) | 0.45 (0.11, 1.79) | 0.31 (0.08, 1.24) | 0.27 (0.06, 1.21) | 0.81 (0.21, 3.17) | 0.65 (0.17, 2.53) |
| 2.38 (0.74, 7.62) | ABA_10 | 1.38 (0.40, 4.69) | 2.68 (0.71, 10.04) | 2.07 (0.55, 7.76) | **5.00 (1.43, 17.53)** | 1.07 (0.27, 4.24) | 0.74 (0.19, 2.92) | 0.65 (0.15, 2.85) | 1.93 (0.49, 7.51) | 1.54 (0.40, 5.99) |
| 1.73 (0.50, 5.92) | 0.73 (0.21, 2.48) | ABA_30/10 | 1.95 (0.49, 7.74) | 1.51 (0.38, 5.98) | 3.64 (0.98, 13.55) | 0.78 (0.19, 3.26) | 0.54 (0.13, 2.25) | 0.47 (0.10, 2.19) | 1.40 (0.34, 5.78) | 1.12 (0.27, 4.61) |
| 0.89 (0.24, 3.34) | 0.37 (0.10, 1.40) | 0.51 (0.13; 2.04) | APR_20 | 0.77 (0.52, 1.15) | **1.87 (1.23, 2.83)** | **0.40 (0.20, 0.81)** | **0.28 (0.14, 0.56)** | **0.24 (0.10, 0.59)** | 0.72 (0.37, 1.40) | 0.57 (0.29, 1.12) |
| 1.15 (0.31, 4.31) | 0.48 (0.13, 1.81) | 0.66 (0.17, 2.63) | 1.29 (0.87, 1.91) | APR_30 | **2.41 (1.60, 3.64)** | 0.52 (0.26, 1.04) | **0.36 (0.18, 0.72)** | **0.31 (0.13, 0.76)** | 0.93 (0.48, 1.81) | 0.74 (0.38, 1.44) |
| 0.48 (0.14, 1.67) | **0.20 (0.06, 0.70)** | 0.28 (0.07, 1.02) | **0.54 (0.35, 0.81)** | **0.41 (0.27, 0.63)** | PLC | **0.21 (0.12, 0.38)** | **0.15 (0.08, 0.26)** | **0.13 (0.06, 0.28)** | **0.39 (0.23, 0.65)** | **0.31 (0.18, 0.52)** |
| 2.22 (0.56, 8.80) | 0.93 (0.24, 3.69) | 1.28 (0.31, 5.37) | **2.50 (1.24, 5.04)** | 1.93 (0.96, 3.89) | **4.67 (2.65, 8.21)** | SEC_075 | 0.69 (0.41, 1.15) | 0.61 (0.29, 1.27) | 1.80 (0.83, 3.89) | 1.44 (0.67, 3.10) |
| 3.22 (0.81, 12.78) | 1.35 (0.34, 5.36) | 1.86 (0.44, 7.80) | **3.63 (1.80, 7.32)** | **2.81 (1.40, 5.65)** | **6.77 (3.85, 11.92)** | 1.45 (0.87, 2.42) | SEC_150 | 0.88 (0.42, 1.85) | **2.61 (1.21, 5.65)** | 2.08 (0.97, 4.50) |
| 3.65 (0.83, 16.10) | 1.54 (0.35, 6.75) | 2.12 (0.46, 9.78) | **4.12 (1.70, 10.01)** | **3.19 (1.32, 7.73)** | **7.69 (3.51, 16.85)** | 1.65 (0.79, 3.46) | 1.14 (0.54, 2.39) | SEC_300 | **2.97 (1.15, 7.62)** | 2.37 (0.92, 6.07) |
| 1.23 (0.32, 4.82) | 0.52 (0.13, 2.02) | 0.71 (0.17, 2.94) | 1.39 (0.71, 2.71) | 1.08 (0.55, 2.09) | **2.59 (1.53, 4.38)** | 0.56 (0.26, 1.20) | **0.38 (0.18, 0.83)** | **0.34 (0.13, 0.87)** | UST_45 | 0.80 (0.48, 1.32) |
| 1.54 (0.40, 6.03) | 0.65 (0.17, 2.53) | 0.89 (0.22, 3.68) | 1.74 (0.89, 3.39) | 1.35 (0.69, 2.62) | **3.25 (1.93, 5.48)** | 0.70 (0.32, 1.50) | 0.48 (0.22, 1.04) | 0.42 (0.16, 1.08) | 1.25 (0.76, 2.07) | UST_90 |
| **A8. ACR20, anti-TNF failure patient population (with anti-TNF experienced population for ABA, UST)** | | | | | | | | | | |
| ABA_03 | 1.02 (0.21, 4.98) | 0.80 (0.20, 3.12) | 0.50 (0.04, 6.01) | 0.58 (0.05, 7.16) | 2.27 (0.36, 14.45) | 1.00 (0.14, 7.29) | 0.78 (0.11, 5.61) | 0.35 (0.04, 2.78) | 0.67 (0.09, 5.17) | 0.73 (0.09, 5.71) |
| 0.98 (0.20, 4.76) | ABA_10 | 0.78 (0.18, 3.36) | 0.49 (0.04, 6.20) | 0.57 (0.04, 7.38) | 2.22 (0.33, 15.18) | 0.98 (0.13, 7.62) | 0.76 (0.10, 5.86) | 0.34 (0.04, 2.90) | 0.65 (0.08, 5.40) | 0.72 (0.09, 5.96) |
| 1.26 (0.32, 4.94) | 1.29 (0.30, 5.56) | ABA_30/10 | 0.63 (0.06, 7.02) | 0.73 (0.06, 8.37) | 2.86 (0.50, 16.43) | 1.26 (0.19, 8.35) | 0.98 (0.15, 6.42) | 0.44 (0.06, 3.20) | 0.84 (0.12, 5.94) | 0.92 (0.13, 6.56) |
| 1.98 (0.17, 23.67) | 2.03 (0.16, 25.55) | 1.58 (0.14; 17.48) | APR_20 | 1.15 (0.34, 3.89) | 4.51 (0.87, 23.49) | 1.99 (0.33, 12.03) | 1.54 (0.26, 9.24) | 0.69 (0.10, 4.64) | 1.32 (0.20, 8.59) | 1.45 (0.22, 9.49) |
| 1.72 (0.14, 21.23) | 1.76 (0.14, 22.89) | 1.37 (0.12, 15.69) | 0.87 (0.26, 2.93) | APR_30 | 3.91 (0.72, 21.40) | 1.73 (0.27, 10.92) | 1.34 (0.21, 8.39) | 0.60 (0.08, 4.20) | 1.15 (0.17, 7.78) | 1.26 (0.19, 8.60) |
| 0.44 (0.07, 2.80) | 0.45 (0.07, 3.07) | 0.35 (0.06, 2.01) | 0.22 (0.04, 1.16) | 0.26 (0.05, 1.40) | PLC | **0.44 (0.21, 0.91)** | **0.34 (0.17, 0.68)** | **0.15 (0.06, 0.40)** | **0.29 (0.12, 0.71)** | **0.32 (0.13, 0.79)** |
| 1.00 (0.14, 7.26) | 1.02 (0.13, 7.94) | 0.79 (0.12, 5.26) | 0.50 (0.08, 3.04) | 0.58 (0.09, 3.67) | **2.27 (1.10, 4.66)** | SEC_075 | 0.78 (0.42, 1.45) | **0.35 (0.14, 0.88)** | 0.67 (0.21, 2.07) | 0.73 (0.23, 2.30) |
| 1.29 (0.18, 9.28) | 1.32 (0.17, 10.15) | 1.02 (0.16, 6.72) | 0.65 (0.11, 3.88) | 0.75 (0.12, 4.68) | **2.92 (1.46, 5.85)** | 1.29 (0.69, 2.41) | SEC_150 | 0.45 (0.18, 1.08) | 0.86 (0.28, 2.63) | 0.94 (0.31, 2.92) |
| 2.89 (0.36, 23.25) | 2.96 (0.34, 25.34) | 2.30 (0.31, 16.92) | 1.46 (0.22, 9.84) | 1.68 (0.24, 11.83) | **6.57 (2.51, 17.19)** | **2.90 (1.13, 7.41)** | 2.25 (0.92, 5.46) | SEC_300 | 1.93 (0.52, 7.10) | 2.12 (0.57, 7.86) |
| 1.50 (0.19, 11.64) | 1.53 (0.19, 12.70) | 1.19 (0.17, 8.46) | 0.76 (0.12, 4.91) | 0.87 (0.13, 5.91) | **3.41 (1.41, 8.22)** | 1.50 (0.48, 4.69) | 1.17 (0.38, 3.58) | 0.52 (0.14, 1.91) | UST_45 | 1.10 (0.52, 2.34) |
| 1.36 (0.18, 10.63) | 1.39 (0.17, 11.59) | 1.08 (0.15, 7.72) | 0.69 (0.11, 4.48) | 0.79 (0.12, 5.39) | **3.10 (1.27, 7.55)** | 1.37 (0.44, 4.29) | 1.06 (0.34, 3.28) | 0.47 (0.13, 1.75) | 0.91 (0.43, 1.93) | UST_90 |
| **A9. ACR20, anti-TNF experienced patient population (with anti-TNF failure population for SEC)** | | | | | | | | | | |
| ABA_03 | 1.02 (0.21, 4.98) | 0.80 (0.20, 3.12) | 0.88 (0.11, 6.92) | 0.66 (0.08, 5.15) | 2.27 (0.36, 14.45) | 1.00 (0.14, 7.29) | 0.78 (0.11, 5.61) | 0.35 (0.04, 2.78) | 0.67 (0.09, 5.17) | 0.73 (0.09, 5.71) |
| 0.98 (0.20, 4.76) | ABA_10 | 0.78 (0.18, 3.36) | 0.86 (0.10, 7.22) | 0.64 (0.08, 5.37) | 2.22 (0.33, 15.18) | 0.98 (0.13, 7.62) | 0.76 (0.10, 5.86) | 0.34 (0.04, 2.90) | 0.65 (0.08, 5.40) | 0.72 (0.09, 5.96) |
| 1.26 (0.32, 4.94) | 1.29 (0.30, 5.56) | ABA_30/10 | 1.11 (0.15, 7.95) | 0.82 (0.11, 5.92) | 2.86 (0.50, 16.43) | 1.26 (0.19, 8.35) | 0.98 (0.15, 6.42) | 0.44 (0.06, 3.20) | 0.84 (0.12, 5.94) | 0.92 (0.13, 6.56) |
| 1.13 (0.14, 8.90) | 1.16 (0.14, 9.71) | 0.90 (0.13; 6.47) | APR_20 | 0.74 (0.35, 1.57) | **2.58 (1.04, 6.38)** | 1.14 (0.36, 3.61) | 0.88 (0.28, 2.76) | 0.39 (0.10, 1.47) | 0.76 (0.21, 2.67) | 0.83 (0.23, 2.96) |
| 1.52 (0.19, 11.97) | 1.56 (0.19, 13.06) | 1.21 (0.17, 8.70) | 1.34 (0.64, 2.83) | APR_30 | **3.47 (1.40, 8.59)** | 1.53 (0.48, 4.87) | 1.19 (0.38, 3.72) | 0.53 (0.14, 1.98) | 1.02 (0.29, 3.60) | 1.12 (0.31, 3.99) |
| 0.44 (0.07, 2.80) | 0.45 (0.07, 3.07) | 0.35 (0.06, 2.01) | **0.39 (0.16, 0.96)** | **0.29 (0.12, 0.72)** | PLC | **0.44 (0.21, 0.91)** | **0.34 (0.17, 0.68)** | **0.15 (0.06, 0.40)** | **0.29 (0.12, 0.71)** | **0.32 (0.13, 0.79)** |
| 1.00 (0.14, 7.26) | 1.02 (0.13, 7.94) | 0.79 (0.12, 5.26) | 0.88 (0.28, 2.80) | 0.65 (0.21, 2.08) | **2.27 (1.10, 4.66)** | SEC_075 | 0.78 (0.42, 1.45) | **0.35 (0.14, 0.88)** | 0.67 (0.21, 2.07) | 0.73 (0.23, 2.30) |
| 1.29 (0.18, 9.28) | 1.32 (0.17, 10.15) | 1.02 (0.16, 6.72) | 1.13 (0.36, 3.55) | 0.84 (0.27, 2.64) | **2.92 (1.46, 5.85)** | 1.29 (0.69, 2.41) | SEC_150 | 0.45 (0.18, 1.08) | 0.86 (0.28, 2.63) | 0.94 (0.31, 2.92) |
| 2.89 (0.36, 23.25) | 2.96 (0.34, 25.34) | 2.30 (0.31, 16.92) | 2.55 (0.68, 9.56) | 1.90 (0.50, 7.11) | **6.57 (2.51, 17.19)** | **2.90 (1.13, 7.41)** | 2.25 (0.92, 5.46) | SEC_300 | 1.93 (0.52, 7.10) | 2.12 (0.57, 7.86) |
| 1.50 (0.19, 11.64) | 1.53 (0.19, 12.70) | 1.19 (0.17, 8.46) | 1.32 (0.37, 4.68) | 0.98 (0.28, 3.48) | **3.41 (1.41, 8.22)** | 1.50 (0.48, 4.69) | 1.17 (0.38, 3.58) | 0.52 (0.14, 1.91) | UST_45 | 1.10 (0.52, 2.34) |
| 1.36 (0.18, 10.63) | 1.39 (0.17, 11.59) | 1.08 (0.15, 7.72) | 1.20 (0.34, 4.28) | 0.89 (0.25, 3.19) | **3.10 (1.27, 7.55)** | 1.37 (0.44, 4.29) | 1.06 (0.34, 3.28) | 0.47 (0.13, 1.75) | 0.91 (0.43, 1.93) | UST_90 |

ABA – Abatacept; ACR – American College of Rheumatology; APR – apremilast; CIs – Confidence intervals; OR – Odds ratio; PASI – Psoriasis Area and Severity Index; PLC – placebo; SEC – secukinumab; UST – ustekinumab

**Table S4. Heterogeneity assessment.**

| Endpoint - time frame - population | Tau^2 | I^2^ | Q value | p value (Q test) |
| --- | --- | --- | --- | --- |
| A1. ACR20, overall population | 0.2939 | 56.7% | 6.53 | 0.163 |
| A2. ACR50, overall population | 0.2756 | 38.3% | 6.34 | 0.175 |
| A3. PASI75, overall population with significant body surface area affected by psoriasis | 0.5434 | 59.4% | 1.85 | 0.763 |
| A4. Any adverse event, overall population | 0 | 0 | 2.69 | 0.847 |
| A5. Severe adverse events, overall population | 0.3663 | 21.2% | 6.82 | 0.338 |
| A6. Withdrawal due to adverse events, overall population | 0 | 0 | 4.40 | 0.623 |
| A7. ACR20, anti-TNF naïve patient population | 0.2264 | 35.6% | 5.68 | 0.460 |
| A8. ACR20, anti-TNF failure patient population (with anti-TNF experienced population for ABA, UST) | 0 | 0 | 0.16 | 0.925 |
| A9. ACR20, anti-TNF experienced patient population (with anti-TNF failure population for SEC) | 0 | 0 | 1.66 | 0.436 |

ACR – American College of Rheumatology; AEs ­– adverse events; PASI – Psoriasis Area and Severity Index; SAEs – Serious adverse events

**Table S5. Average probability of an outcome (95% CIs).**

|  | A1. ACR20, overall population | A2. ACR50, overall population | A3. PASI75, overall population | A4. Any adverse event | A5. Severe adverse events | A6. Withdrawal due to adverse events | A7. ACR20, anti-TNF naïve patient**s** | A8. ACR20, anti-TNF failure patient**s** | A9. ACR20, anti-TNF experienced patient**s** |
| --- | --- | --- | --- | --- | --- | --- | --- | --- | --- |
| ABA_03 | 31.9% (13.0%, 59.5%) | 32.8% (5.1%, 81.6%) | 45.5% (6.8%, 90.5%) | 51.3% (29.6%, 72.6%) | 1.1% (0.0%, 22.9%) | 1.3% (0.1%, 11.5%) | 35.7% (13.6%, 66.2%) | 26.1% (5.3%, 69.2%) | 26.7% (5.4%, 69.9%) |
| ABA_10 | 45.9% (21.3%, 72.7%) | 46.9% (9.1%, 88.6%) | 18.4% (1.7%, 74.9%) | 62.1% (37.7%, 81.7%) | 6.0% (0.7%, 36.6%) | 2.9% (0.5%, 16.0%) | 56.9% (27.4%, 82.2%) | 25.6% (4.8%, 70.2%) | 26.3% (5.0%, 70.9%) |
| ABA_30/10 | 40.3% (17.8%, 67.8%) | 41.2% (7.3%, 86.2%) | 13.1% (1.0%, 69.2%) | 49.7% (28.1%, 71.3%) | 10.1% (1.5%, 46.2%) | 1.3% (0.1%, 12.0%) | 49.0% (20.5%, 78.2%) | 30.7% (7.2%, 71.8%) | 31.4% (7.4%, 72.5%) |
| APR_20 | 31.1% (22.4%, 41.3%) | 14.1% (8.6%, 22.3%) | 22.2% (10.1%, 42.0%) | 67.4% (61.6%, 72.7%) | 3.2% (1.4%, 6.9%) | 5.5% (3.1%, 9.4%) | 33.0% (24.6%, 42.7%) | 41.2% (11.8%, 78.5%) | 29.2% (14.3%, 50.6%) |
| APR_30 | 36.2% (26.8%, 46.9%) | 15.1% (9.3%, 23.7%) | 25.2% (11.8%, 46.0%) | 67.1% (61.3%, 72.4%) | 3.4% (1.6%, 7.1%) | 7.1% (4.2%, 11.9%) | 38.9% (29.7%, 49.0%) | 37.8% (10.0%, 76.9%) | 35.7% (18.3%, 57.9%) |
| SEC_075 | 44.2% (30.9%, 58.4%) | 22.0% (12.9%, 34.9%) | 32.9% (15.5%, 56.8%) | 52.5% (44.4%, 60.5%) | 2.9% (1.0%, 7.9%) | 3.2% (1.2%, 8.5%) | 55.2% (41.2%, 68.4%) | 26.0% (14.6%, 41.9%) | 26.7% (15.1%, 42.8%) |
| SEC_150 | 53.5% (39.5%, 67.0%) | 30.7% (19.0%, 45.5%) | 39.6% (19.9%, 63.4%) | 58.5% (50.4%, 66.1%) | 3.0% (1.1%, 8.1%) | 2.0% (0.6%, 6.7%) | 64.2% (50.4%, 75.9%) | 31.2% (18.5%, 47.6%) | 31.9% (19.0%, 48.4%) |
| SEC_300 | 61.7% (43.0%, 77.6%) | 34.1% (18.5%, 54.1%) | 55.3% (25.1%, 82.0%) | 56.6% (44.3%, 68.1%) | 5.8% (1.4%, 21.3%) | 3.3% (0.7%, 13.9%) | 67.0% (48.1%, 81.7%) | 50.5% (28.0%, 72.7%) | 51.3% (28.7%, 73.4%) |
| UST_45 | 40.3% (22.5%, 61.1%) | 15.9% (6.1%, 35.5%) | 57.5% (22.7%, 86.2%) | 59.9% (52.0%, 67.4%) | 2.7% (0.8%, 8.7%) | 1.2% (0.4%, 3.2%) | 40.7% (28.9%, 53.7%) | 34.6% (18.0%, 56.1%) | 35.3% (18.5%, 56.9%) |
| UST_90 | 40.5% (22.6%, 61.2%) | 21.0% (8.6%, 42.9%) | 61.7% (25.9%, 88.1%) | 57.9% (49.9%, 65.4%) | 1.8% (0.6%, 5.5%) | 1.4% (0.5%, 3.5%) | 46.2% (33.8%, 59.1%) | 32.5% (16.5%, 53.9%) | 33.2% (16.9%, 54.8%) |
| PLC (assumed control rate) | 18.1% (15.6%, 20.6%) | 6.1% (4.6%, 7.7%) | 6.4% (4.0%, 9.1%) | 54.4% (49.0%, 59.7%) | 3.5% (2.4%, 4.7%) | 4.2% (2.6%, 6.1%) | 20.9% (18.2%, 23.7%) | 13.4% (8.7%, 18.9%) | 13.8% (9.5%, 18.7%) |

ABA – abatacept; ACR – American College of Rheumatology; AEs – Adverse events; APR – apremilast; CIs – Confidence intervals; OR – Odds ratio; PASI – Psoriasis Area and Severity Index; PLC – placebo; SAEs – Serious adverse eve; SEC – secukinumab; UST – ustekinumab

**Table S6.** **The total sample sizes for hypothetical between-treatment comparative studies (likelihood-ratio test, 80% power, 2-sided alpha of 0.05).**

| Treatment 1 | Treatment 2 | A1. ACR20, overall population | A7. ACR20, anti-TNF naïve patient population | A8. ACR20, anti-TNF failure patient population | A9. ACR20, anti-TNF experienced patient population |
| --- | --- | --- | --- | --- | --- |
| ABA_03 | ABA_10 | **382** | **174** | 324,376 | 319,296 |
| ABA_03 | ABA_30/10 | 1,030 | **436** | 2,958 | 2,916 |
| ABA_03 | APR_20 | 104,306 | 9,598 | **306** | 9,864 |
| ABA_03 | APR_30 | 3,776 | 7,234 | **496** | **828** |
| ABA_03 | SEC_075 | **488** | **204** | 41,488,018 | 40,844,762 |
| ABA_03 | SEC_150 | **164** | **96** | 2,430 | 2,396 |
| ABA_03 | SEC_300 | **88** | **80** | **124** | **122** |
| ABA_03 | UST_45 | 1,018 | 3,058 | **912** | **900** |
| ABA_03 | UST_90 | **990** | **694** | 1,584 | 1,562 |
| ABA_10 | ABA_30/10 | 2,460 | 1,248 | 2,466 | 2,430 |
| ABA_10 | APR_20 | **340** | **136** | **288** | 7,136 |
| ABA_10 | APR_30 | **814** | **242** | **460** | **750** |
| ABA_10 | SEC_075 | 28,160 | 26,654 | 390,356 | 384,238 |
| ABA_10 | SEC_150 | 1,356 | 1,432 | 2,058 | 2,030 |
| ABA_10 | SEC_300 | **310** | **724** | **120** | **118** |
| ABA_10 | UST_45 | 2,506 | **296** | **822** | **812** |
| ABA_10 | UST_90 | 2,616 | **682** | 1,384 | 1,366 |
| ABA_30/10 | APR_20 | **852** | **298** | **662** | 13,984 |
| ABA_30/10 | APR_30 | 4,496 | **762** | 1,416 | 3,782 |
| ABA_30/10 | SEC_075 | 4,950 | 2,030 | 2,908 | 2,868 |
| ABA_30/10 | SEC_150 | **448** | **336** | 276,644 | 273,240 |
| ABA_30/10 | SEC_300 | **170** | **234** | **194** | **192** |
| ABA_30/10 | UST_45 | 28,976,772 | 1,118 | 4,578 | 4,526 |
| ABA_30/10 | UST_90 | 2,660,222 | 10,000 | 21,932 | 21,670 |
| APR_20 | APR_30 | 2,666 | 2,074 | 6,536 | 1,638 |
| APR_20 | SEC_075 | **428** | **156** | **304** | 9,564 |
| APR_20 | SEC_150 | **152** | **80** | **730** | 9,302 |
| APR_20 | SEC_300 | **82** | **68** | **900** | **154** |
| APR_20 | UST_45 | **844** | 1,252 | 1,712 | 1,840 |
| APR_20 | UST_90 | **824** | **432** | **966** | 4,302 |
| APR_30 | SEC_075 | 1,180 | **294** | **494** | **822** |
| APR_30 | SEC_150 | **260** | **122** | 1,642 | 4,856 |
| APR_30 | SEC_300 | **120** | **98** | **480** | **318** |
| APR_30 | UST_45 | 4,386 | 24,938 | 7,176 | 510,918 |
| APR_30 | UST_90 | 4,148 | 1,450 | 2,544 | 11,146 |
| SEC_075 | SEC_150 | **912** | **946** | 2,394 | 2,360 |
| SEC_075 | SEC_300 | **254** | **534** | **124** | **122** |
| SEC_075 | UST_45 | 5,082 | **370** | **902** | **892** |
| SEC_075 | UST_90 | 5,406 | **966** | 1,564 | 1,544 |
| SEC_150 | SEC_300 | 1,128 | 8,564 | **204** | **202** |
| SEC_150 | UST_45 | **452** | **142** | 6,028 | 5,962 |
| SEC_150 | UST_90 | **460** | **240** | 42,486 | 41,988 |
| SEC_300 | UST_45 | **172** | **112** | **304** | **302** |
| SEC_300 | UST_90 | **172** | **178** | **234** | **234** |
| UST_45 | UST_90 | 5,475,760 | 2,516 | 15,508 | 15,344 |

ABA – abatacept; ACR – American College of Rheumatology; APR – apremilast; PLC – placebo; SEC – secukinumab; UST – ustekinumab
