# Supplementary material for: Comparative effectiveness of abatacept, apremilast, secukinumab and ustekinumab treatment of psoriatic arthritis: a systematic review and network meta-analysis
Source: Rheumatol Int. 2017 Dec 28;38(2):189–201. doi: 10.1007/s00296-017-3919-7 (PMC5773655; doi:10.1007/s00296-017-3919-7)
Supplement: Supplementary file 2 — Supplementary material 2 (DOCX 104 KB) [file 296_2017_3919_MOESM2_ESM.docx]

**Network graphs by endpoint.**

| 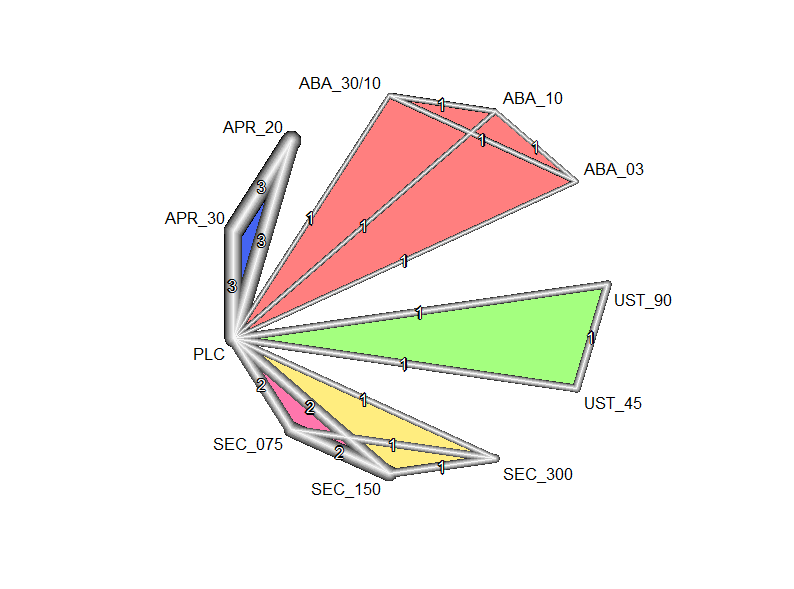ACR20, overall population  ACR50, overall population  PASI75, overall population affected by psoriasis | 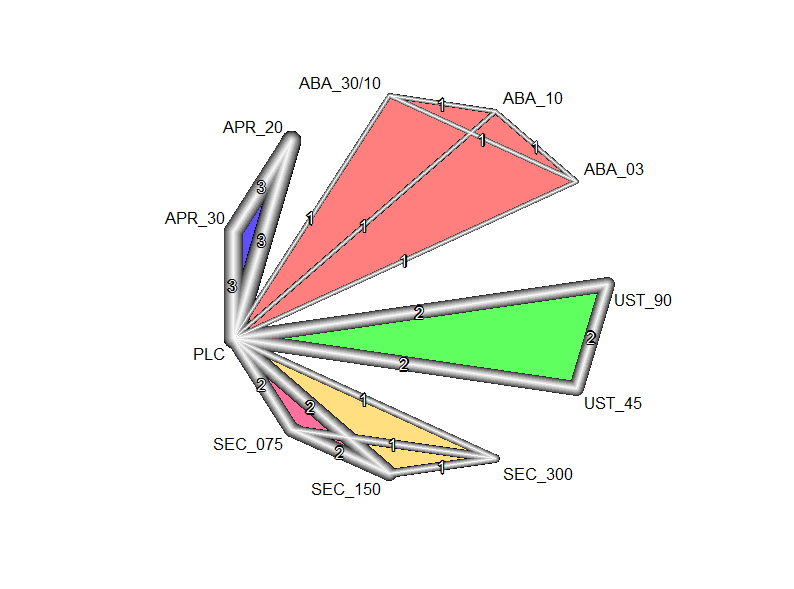Any AE, overall population  SAEs, overall population  Withdrawal due to AEs, overall population  ACR20, anti–TNF-α-naive patient population |
| --- | --- |
| 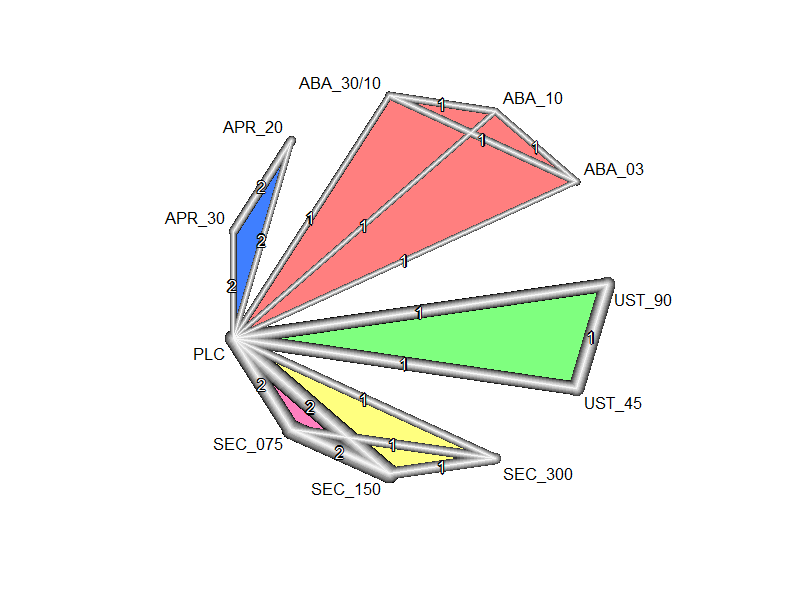ACR20, anti–TNF-α-failure population^a^  ACR20, anti–TNF-α-experienced population^b^ |  |

^a^ With supplementation of results from anti–TNF-α-experienced population for abatacept and ustekinumab.

^b^ With supplementation of results from anti–TNF-α-failure population for secukinumab.

Abbreviations: ABA – abatacept; ACR – American College of Rheumatology; AE, adverse event; APR – apremilast; PASI*,* Psoriasis Area and Severity Index; PLC – placebo; SAE, severe adverse event; SEC – secukinumab; UST – ustekinumab.
